# TRPS1 regulates the opposite effect of progesterone via RANKL in endometrial carcinoma and breast carcinoma

**DOI:** 10.1038/s41420-023-01484-0

**Published:** 2023-06-21

**Authors:** Linlin Yang, Qiong Fan, Jing Wang, Xiaoming Yang, Jiangjing Yuan, Yuhong Li, Xiao Sun, Yudong Wang

**Affiliations:** 1 Department of Gynecological Oncology, The International Peace Maternity and Child Health Hospital, School of Medicine, Shanghai Jiao Tong University, Shanghai, China; 2 Shanghai Municipal Key Clinical Specialty, Shanghai, China; 3 Shanghai Key Laboratory of Embryo Original Disease, Shanghai, China

**Keywords:** Tumour-suppressor proteins, Cell growth

## Abstract

Medroxyprogesterone (MPA) has therapeutic effect on endometrial carcinoma (EC), while it could promote the carcinogenesis of breast cancer (BC) by activating receptor activator of NF-kB ligand (RANKL). However, the selective mechanism of MPA in endometrium and breast tissue remains obscure. Multiomics analysis of chromatin immunoprecipitation sequencing (ChIP-seq) and RNA sequencing (RNA-seq) were performed in cell lines derived from endometrial cancer and mammary tumor to screen the differential co-regulatory factors of progesterone receptor (PR). Dual-luciferase assays and ChIP-PCR assays were used to validate the transcriptional regulation. Co-immunoprecipitation (Co-IP) and immunofluorescence assays were carried out to explore molecular interactions between PR, the cofactor transcriptional repressor GATA binding 1 (TRPS1), and histone deacetylase 2 (HDAC2). Subsequently, human endometrial cancer/breast cancer xenograft models were established to investigate the regulation effect of cofactor TRPS1 in vivo. In the current study, we found that MPA downregulated RANKL expression in a time- and dose-dependent manner in EC, while had the opposite effect on BC. Then PR could recruit cofactor TRPS1 to the promoter of RANKL, leading to histone deacetylation of RANKL to repress its transcription in EC, whereas MPA disassociated the PR/TRPS1/HDAC2 complex to enhance RANKL histone acetylation in BC. Therefore, TRPS1, the coregulator recruited by PR played a critical role in the selective mechanism of progesterone in EC and BC and could become a potential candidate for targeted therapy to improve the anticancer effect of MPA on EC and avoid its carcinogenic effect on BC.

## Introduction

Endometrial cancer (EC) is one of the most common gynecologic malignancies worldwide [1], with an extensively growing morbidity and mortality, especially among younger women [2]. There are an estimated 65950 new cancer cases and 12,550 cancer deaths diagnosed in uterine corpus in the United States 2022 [3]. About 3–14% of EC cases are found in premenopausal women under 40 years old who have a strong desire to preserve their fertility [4], for whom the standard care of hysterectomy in combination with bilateral salpingo-oophorectomy is not suitable [5]. Up to now, as an alternative to hysterectomy, progestin therapy is widely utilized for conservative management of EC patients [6]. Clinical researchers reported that the response rate of progestin treatment in patients with early-stage EC or precancerous lesions was only approximately 70%, the remaining 30% failed to respond [7, 8], even among women with a complete response, almost 35–40% cases ultimately recurred [9, 10]. The reasons for this failure are still elusive. Progestin insensitivity remains a major blockage for administering conservative therapy for endometrial cancer patients.

Female breast cancer incidence rates have been increasing by about 0.5% per year since the mid-2000s with 287,850 new cases and 43,250 estimated deaths in 2022 [3]. Principal results from the Women’s Health Initiative randomized controlled trial [11], Million Women Study [12], E3N-EPIC cohort [13], and a case–control study in Finland [14] demonstrated that synthetic progestin used to treat endometrial cancer contributed to the initiation and/or progression of breast cancer. Compared with estrogen only therapy (1.15, 95% confidence interval 1.09–1.21), combined progestogens’ therapy was associated with increased risks of breast cancer (1.88, 95% confidence interval 1.79–1.99) [15]. Thus, progesterone was listed as a suspected carcinogen.

The RANKL/RANK system, which is a member of TNF superfamily, regularizes bone differentiation and maturation by soluble agents and homogeneous interaction, which facilitates bone reabsorption [16]. Emerging evidence suggests that RANKL/RANK pathway is not limited to bone remodeling, RANKL exactly acts as a multifunctional cytokine that is indispensable for the formation of carcinomas [17], such as endometrial cancer [18] and breast cancer (BC) [19]. According to our previous studies, the expression of RANKL was upregulated in EC tissues and administration of medroxyprogesterone acetate (MPA) was able to inhibit the EC cell behavior induced by RANKL via progesterone receptor (PR) [20], while in BC, treatment with MPA triggered massive induction of RANKL in luminal epithelial cells, leading to increased proliferation of mammary epithelial cells, thus giving rise to mammary tumor in combination with a chemical carcinogen, 7,12-dimethylbenz[a]anthracene (DMBA) in vivo [21]. Based on the differential regulation of RANKL by MPA between endometrial and breast tissues, its underlying selective regulation mechanisms remain to be explored in detail.

On the basis of profound researches [22, 23], Shang illuminated that tamoxifen, as a selective estrogen receptor (ER) modulator, stimulated the recruitment of coactivators of ER to a subset of genes in EC, while induced the recruitment of corepressors to target gene promoters in mammary cells, hence exerting its selective action in different cell types. Therefore, we hypothesized that whether PR could recruit coregulators to execute the selective effects of MPA on RANKL expression.

In this study, we set out to elucidate the selective regulation mechanism of MPA in EC and BC. Our results identified TRPS1 as a novel cofactor of PR-dependent recruitment by specifically binding to HDAC2, a member of corepressor complex, changing acetylation levels of the target gene RANKL, inducing its different expressions in different cancer cells. This finding not merely provided key insights into the complex context-dependent mechanisms of PR coregulators, but also supported the pursuit of TRPS1 as the potential therapeutic target of endometrial carcinoma.

## Results

### Identification of the differentially expressed genes in EC and BC cells

**Fig. 1:**
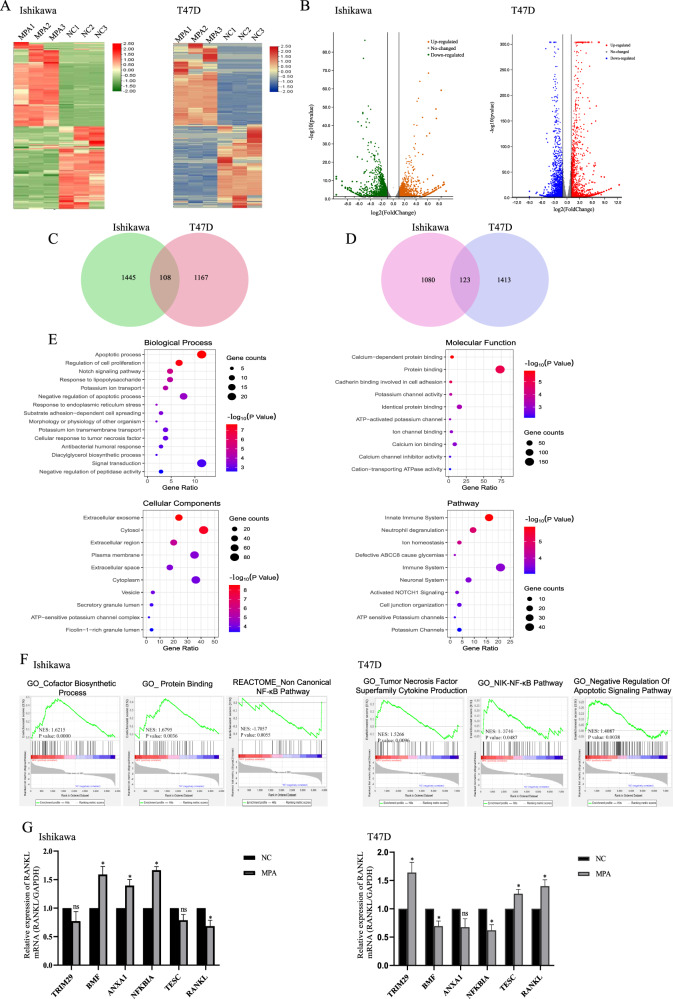
MPA treatment changed the genic signature of endometrial cancer and breast cancer cells. **A** Heatmap of differentially expressed genes in Ishikawa and T47D cells treated with MPA (20 μM for 48 h) versus DMSO. **B** Volcano plot showing the differentially expressed genes (DEGs) between the MPA-treated and DMSO groups. **C** Venn diagram to identify the common genes of significantly downregulated transcripts in Ishikawa and the significantly upregulated transcripts inT47D cells after treatment with 20 μM MPA for 48 h. **D** Venn diagram of the significantly upregulated transcripts in Ishikawa and the significantly downregulated transcripts in T47D cells after MPA treatment. **E** GO and pathway analyses of the 108 overlapping significantly altered common genes. **F** GSEA showing the enrichment of gene sets in DMSO- or MPA-treated Ishikawa and T47D cell lines. **G** The validation experiments by RT-PCR in Ishikawa and T47D cells. *p < 0.05. ns not significant.

To investigate the transcriptome profile induced by MPA treatment in EC and BC, respectively, we incubated endometrial cancer cell line Ishikawa and breast cancer cell line T47D with 20 μM MPA for 48 h to conduct RNA sequencing and screened differentially expressed genes (DEGs) using the limma R package (log_2_|FC| > 1 and *p* value < 0.05). There were 2756 DEGs between MPA treatment group and non-treatment group in Ishikawa, meanwhile, 2811 DEGs were found in T47D (Supplementary Materials 1 and 2). The heatmap of the gene expression profile was shown in Fig. 1A and the volcano plot of all the genes detected was displayed in Fig. 1B. Under our experimental conditions, a total of 108 hub DEGs (1553 genes were downregulated in Ishikawa cells, 1275 genes were upregulated in T47D cells) and 123 common genes (1203 genes were upregulated in Ishikawa, 1536 genes were downregulated in T47D) were revealed in two cell lines, respectively (Fig. 1C, D). The overall 108 DEGs information was saved for further analysis (Supplementary Material 3).

Gene Ontology (GO) functional annotation and Reactome pathway annotation of the identified 108 common genes was obtained using the DAVID online analysis tool (https://david.ncifcrf.gov/). The results were deemed statistically significant at a cutoff of FDR < 0.01. The top 15 GO and Reactome pathway terms of the common genes were depicted in Fig. 1E and they were mainly enriched in cellular response to tumor necrosis factor, regulation of cell proliferation and apoptotic process. The detailed differential pathway analysis in each cell lines was shown in Supplementary Fig. 1. Furthermore, gene set enrichment analysis (GSEA) was utilized to identify, characterize, and link potential biological pathways involved in the effects of MPA in carcinomas. Utilizing statistical cutoffs of a *p* value < 0.05, we identified a series of tumor necrosis factor superfamily cytokine production, NF-κB pathway and cofactor biosynthetic process-associated gene sets that were aberrantly altered in the MPA-treated group compared to the DMSO control group (Fig. 1F). Moreover, to verify the accuracy and reliability of sequencing, we adopted quantitative real-time PCR (qRT-PCR) to detect the transcription of the remarkably altered six common genes in both cell lines. We found that the mRNA levels of BMF, ANXA1 and NFκB1A were significantly increased and RANKL transcription was reduced in MPA-treated Ishikawa cells, while the expressions of TRIM29 and TESC were not statistically significant. In T47D cells, the levels of TRIM29, TESC, RANKL were validated to be elevated, BMF and NFκB1A transcriptions were decreased after MPA treatment, whereas the mRNA expression of ANXA1 was not substantially repressed compared with control group (Fig. 1G). The RANKL gene (Receptor activator of nuclear factor-κB ligand), TNF superfamily member 11, encoded the member of the tumor necrosis factor (TNF) cytokine family, coinciding with the enriched pathway analysis of differential common genes and attracting our attention for further exploration.

### The crucial role of the aberrant RANKL in MPA-pretreated endometrial cancer and breast cancer cells

**Fig. 2:**
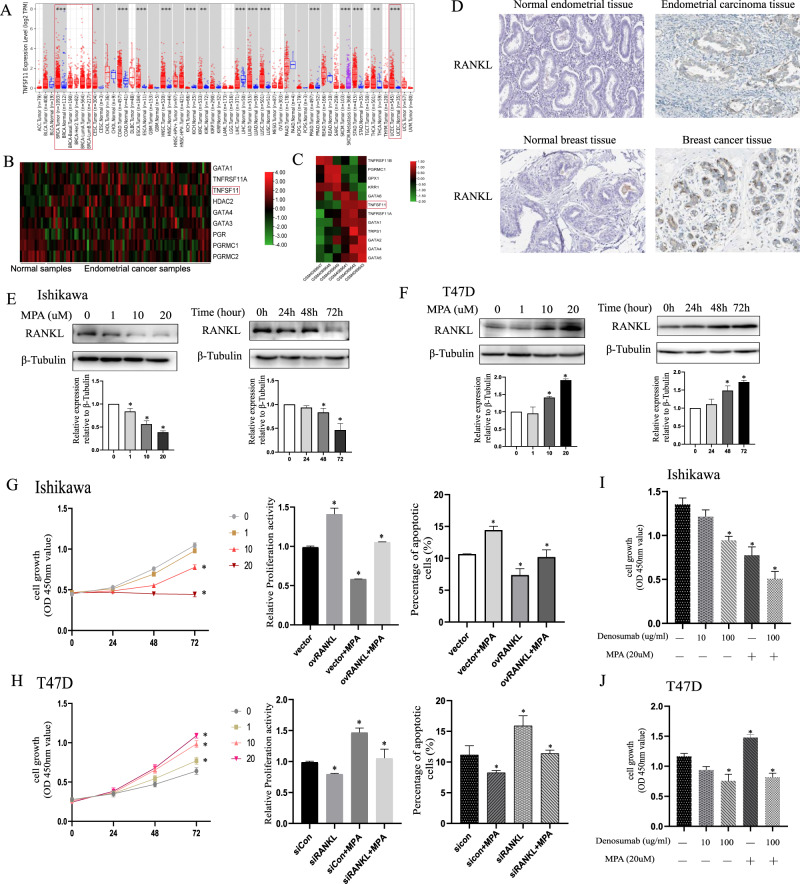
Aberrant expression of RANKL played a essential role in the selective regulation of MPA in endometrial cancer and breast cancer. **A** Analysis of the expression of RANKL in different tumors or specific cancer subtypes by TIMER2. **B** Heatmap of RANKL expression in EC tissue based on GSE17025. **C** Heatmap of RANKL expression in BC tissue according to GSE137842. **D** Immunohistochemical staining for RANKL expression in normal and cancer lesion tissues of endometrium and mammary gland, respectively. **E** A gradient of MPA was applied to Ishikawa to detect RANKL expression at the indicated time points by western blotting assays. **F** T47D cells were treated with different concentrations of MPA and harvested at indicated time points for evaluation of RANKL protein levels by western blotting. The intensity of RANKL was normalized to the intensity of β-tubulin by Image J. **G** Treatment with a gradient of MPA affected cell growth and apoptosis and overexpression of RANKL impaired this effect on Ishikawa. **H** T47D cells were treated with different doses of MPA with or without RANKL silencing, then we detected cell activity and cell apoptosis by CCK8 assays and flow cytometry, respectively. **I**, **J** CCK8 assays were implemented to evaluate the effect of denosumab in the absence or presence of MPA on Ishikawa and T47D cells, respectively. *p < 0.05, **p < 0.01, ***p < 0.001.

RANKL and its receptor RANK (receptor activator of nuclear factor-kappa B) were primarily discovered within the immune and the bone systems [24]. Recently, interest has increased in the role of the RANKL system in tumorigenesis [25]. We first analyzed RANKL expression among different tumors in the TCGA database utilizing the TIMER2 method (Fig. 2A). We observed a statistically significant overexpression in breast invasive carcinoma (BRCA) and uterine corpus endometrial carcinoma (UCEC) compared with the relevant adjacent normal tissues. Next, we searched the TCGA database and found that the rate of mutation of RANKL was less than 2% in all enrolled endometrial cancer cases (Supplementary Fig. 2A, B) and its mutation rate was not profiled in breast carcinoma (Supplementary Fig. 2C). Then, gene expression profiling data sets obtained from the GEO database were analyzed. In GSE17025, which was comprised of ninety-one samples of stage I human endometrial cancers (79 endometrioid and 12 serous) and twelve normal endometrium tissue specimens from surgery [26], the expression of RANKL was 2.15 times higher in endometrioid cancer than in normal tissues (Fig. 2B). Likewise, in GSE137842, which was derived from breast cancer cells [27], the expression profile of RANKL was 5.9 times higher in primary mammary tumors that metastasized to bone than that did not metastasize (Fig. 2C). By immunohistochemistry (IHC) analysis on endometrial tissues, RANKL staining was predominantly localized to the cytoplasmic membrane, weak or no staining was detected in the normal endometrium, and moderate to strong RANKL staining was found in endometrial adenocarcinoma. In breast tissues, more intense and abundant levels of RANKL protein distributed in the cytoplasm and intercellular matrix in cancer tissues (Fig. 2D).

Although previous evidence demonstrated that progesterone treatment attenuated the carcinogenic effect of RANKL [20], the underlying regulatory mechanism warranted further elucidated. In the current study, MPA treatment resulted in a potent decrease of RANKL expression, which occurred in a dose- and time-dependent manner in endometrial cancer cell lines (Fig. 2E). Interestingly, in breast cancer cells, the protein expression of RANKL was enhanced after MPA treatment in the similar dose- and time-dependent manner (Fig. 2F and Supplementary Fig. 4A). Cell growth assays indicated that MPA treatment significantly inhibited the growth activity of endometrial cancer (Fig. 2G). Taking into consideration that RANKL was reported to be correlated with the mediation of MPA in cells [20], we then upregulated RANKL expression through plasmid transfection of RANKL in cells (Supplementary Fig. 3A). The findings revealed that RANKL overexpression impaired the inhibitory effect of MPA on cell proliferation and cell apoptosis in EC cells (Fig. 2G and Supplementary Fig. 4B). Additionally, we depleted RANKL expression by transfecting with two independent siRNAs in cells, the transfection efficiency was evaluated by RT-PCR and western blotting assays (Supplementary Fig. 3B) and siRANKL-2 was selected for subsequent experiments for its highest knockdown efficiency. The results indicated that MPA-triggered cell activity was alleviated and cell apoptotic percentage was increased in BC cells after RANKL knockdown (Fig. 2H and Supplementary Fig. 4C). Moreover, we uncovered that RANKL silencing in Ishikawa could inhibit cell growth and promote cell apoptosis as well (Supplementary Fig. 5A) and overexpression of RANKL in T47D could enhance MPA-induced cell proliferation and decrease cell apoptosis (Supplementary Fig. 5B), thus further emphasizing the essential role of RANKL in the selective regulation of progesterone in EC and BC cells.

Denosumab, a humanized monoclonal RANKL antibody, which could play a crucial role in different physiological activity including the prevention of skeletal-related events arising from cancer [28], was FDA approved for breast cancer, prostate cancer and so on [29], but had not been studied in endometrial cancer. We used a gradient of increasing doses of denosumab to act on cancer cells and revealed that in line with previous researches, denosumab more profoundly decreased MPA-induced cell activity in T47D, while it could enhance the growth inhibition of MPA on endometrial cancer cells (Fig. 2I, J), highlighting that denosumab may exert a potential antitumor effect on EC.

### The mediation of RANKL by MPA was PR dependent

**Fig. 3:**
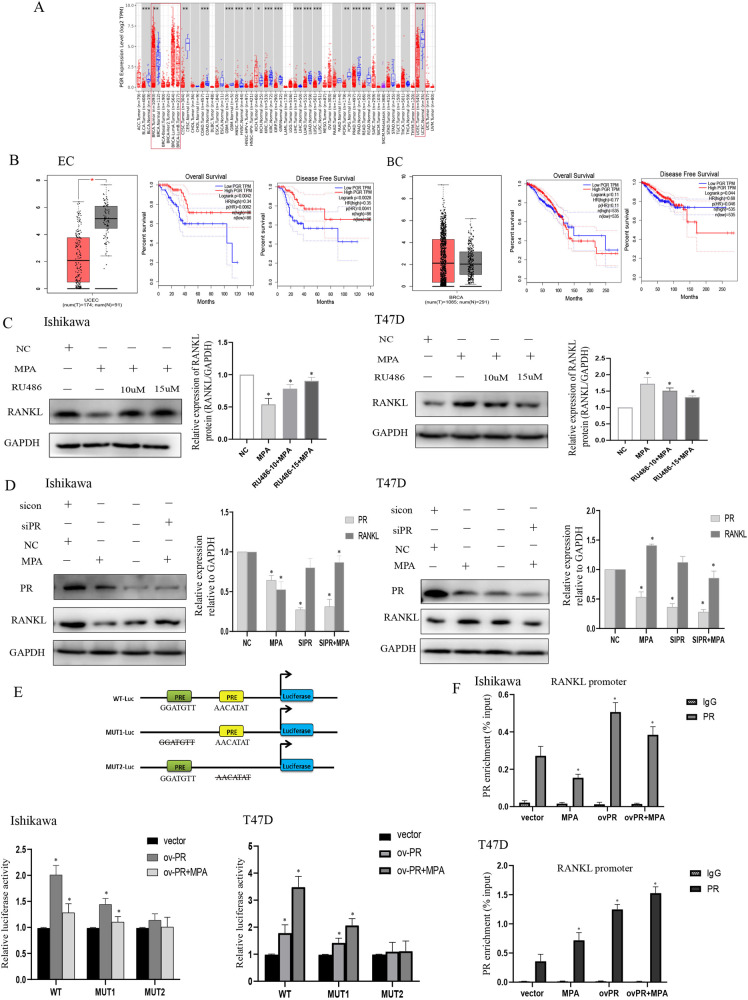
MPA regulated the expression of RANKL via its receptor PR. **A** Analysis of the expression of PR in different tumors by TIMER2 database. **B** The expression and survival analysis of PR in endometrial tissues and breast tissues by GEPIA, respectively. **C** Western blotting showing the levels of RANKL in Ishikawa and T47D cells treated with RU486 or in combination with MPA. **D** Silencing of PR attenuated the effect of MPA on RANKL expression by Western blotting analysis. **E** Potential PR binding site in the promoter of RANKL examined with dual-luciferase reporter assay by transfecting the wild-type (WT) or mutated (MUT1, MUT2) plasmids. **F** Binding of PR to the predicted site was confirmed by ChIP-PCR using primers specific to the binding site in Ishikawa and T47D cells. *p < 0.05, **p < 0.01, ***p < 0.001.

Progestins mainly exerted their effect through binding to PR, we first explored the PR expression in different tumors by TIMER2 database. The results indicated that compared with normal endometrial tissues, PR was downregulated in UCEC. Although distinct subtypes of BRCA presented different levels of PR, in a whole, the expression of PR was lower in BRCA tumor tissues than that in normal tissues (Fig. 3A). Likewise, according to GEPIA, the expression of PR was decreased in EC tissues and higher PR expression was associated with worse overall survival (OS) and disease-free survival (DFS). Whereas, there was no significant difference of PR expression between breast cancer tissues and normal tissues and it was not related to OS, but higher PR expression was linked to a worse disease-free survival in BC (Fig. 3B). RU486, a PR antagonist, alleviated the down-regulation effect of MPA on RANKL in Ishikawa and also impaired the up-regulation effect of MPA on RANKL in T47D (Fig. 3C). Additionally, transfection of siPR significantly downregulated PR expression, also blocked the inhibition of MPA on RANKL in Ishikawa and suppressed the induction of MPA on RANKL in T47D (Fig. 3D), suggesting that MPA regulated RANKL expression through its receptor PR.

To understand the molecular mechanisms by which MPA/PR modulated RANKL expression, we analyzed the RANKL sequence and found two common PRE (site 1: GGATGTT; site 2: AACATAT) in the promoter region of RANKL gene (Fig. 3E). We constructed three reporter plasmids (WT, MUT1, MUT2) in which truncated RANKL regulatory regions were positioned upstream of a luciferase gene to determine which binding site was involved. The wild-type (WT), mutated 1 (MUT1), or mutated 2 (MUT2) promoter vectors plus a plasmid overexpressing PR or its negative control were co-transfected into cells with or without MPA treatment to perform dual-luciferase reporter assays. We observed that overexpression of PR potently enhanced luciferase activity after co-transfecting the WT plasmid, while the luciferase activity was significantly lower when co-transfecting the MUT1 plasmid, and after MPA treatment the activity was decreased in both groups. However, no changes were detected following co-transfection with PR-overexpressing plasmid plus the MUT2 plasmid in the presence or absence of MPA in Ishikawa. The luciferase activity of T47D cells was increased more significantly in the group that co-transfected with the WT plasmid than that co-transfected with MUT1 plasmid, and the activity was further induced in the presence of MPA. Similarly, no changes were observed following co-transfection with PR together with the MUT2 plasmid with or without using MPA (Fig. 3E). These results suggested that MPA/PR regulated RANKL transcription via binding to the specific PRE site in the promoter region in different cell lines.

On the basis of previous studies, a region (−2200/−1000 bp) of RANKL promoter had been identified to display strong PR-binding activity and was enriched with DNA-binding motifs, which were important for PR recruitment to DNA [30–32]. We then determined whether PR was recruited to the promoter region in cancer cells using ChIP-qPCR. The results revealed that PR enrichment in the RANKL promoter (−1256/−1118 bp) was markedly enhanced in both EC and BC cells after transfection with PR. Additionally, MPA treatment weakened the binding of PR to the promoter in Ishikawa, but on the other hand, it further facilitated the enrichment of PR in T47D cells (Fig. 3F), suggesting that there may exist potential mechanisms which could illustrate the differential expression regulation of RANKL in different cell contexts in the presence of MPA.

### Cofactor TRPS1 was correlated with PR in cancer cells

**Fig. 4:**
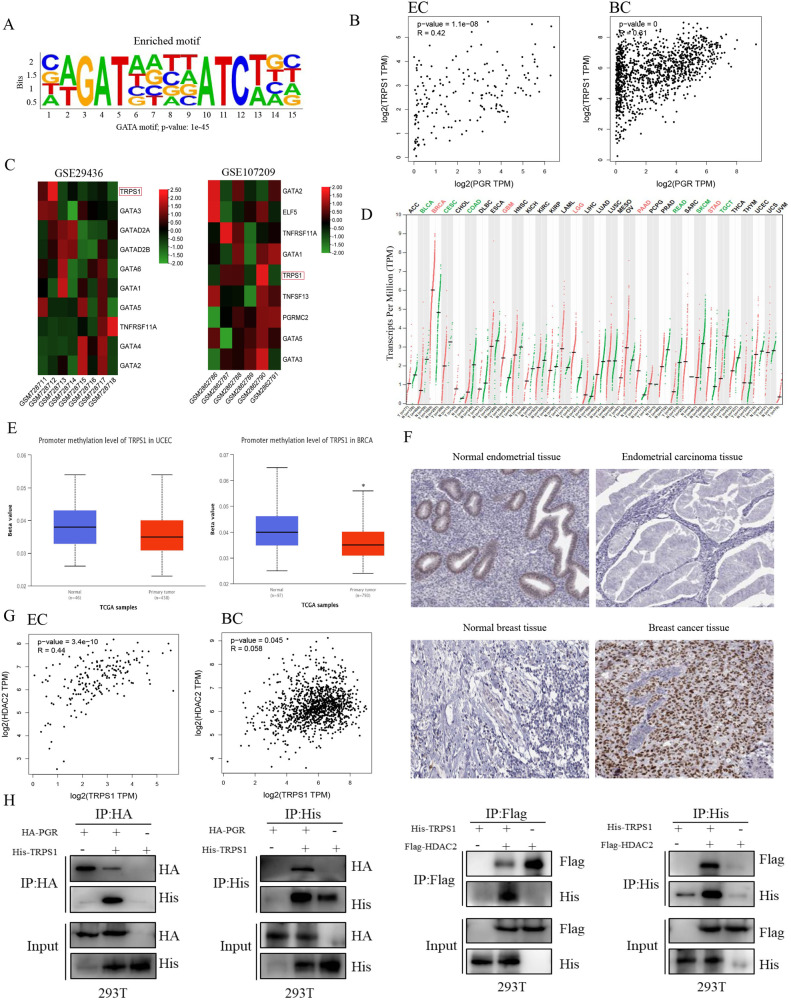
TRPS1 may be involved in forming a PR/TRPS1/HDAC2 complex to exert its effect on cancer cells. **A** The present of the enriched motif of PR by ChIP-seq assay. **B** The correlation between PR and TRPS1 was explored by GEPIA database in EC and BC, respectively. **C** Heatmap of GATA family members in EC based on GSE29436 and heatmap of GATA family members in BC according to GSE107209. **D** Exploration of the expression of TRPS1 in different kinds of tumors by GEPIA database. Each dots represented the expression of samples. **E** Promoter methylation level of TRPS1 in normal and tumor tissues of endometrium and mammary gland in TCGA dataset, respectively. **F** Immunohistochemical analysis of TRPS1 expression in normal and tumor samples of endometrium and mammary gland, respectively. **G** The mutual correlation between TRPS1 and HDAC2 was investigated by GEPIA website in EC and BC, respectively. **H** HEK-293T cells were co-transfected with HA-PGR and His-TRPS1, or His-TRPS1 and Flag-HDAC2 for 36 h and cell lysates were prepared for co-IP assays. *p < 0.05.

It is well acknowledged that PR often functions as a transcriptional factor, could recruit pro-regulatory proteins (coactivators or corepressors) in the nucleus, thus interacting with the transcription apparatus to modulate gene expression, promoting diverse cellular functions [33–35]. Based on above findings, we hypothesized that PR might recruit regulatory cofactors to form a functional machinery to suppress or activate gene expression, thus resulting in the selective regulation of MPA in different cells. From ChIP-Seq assay, HOMER motif analysis identified GATA binding motif as the most significant enriched motif (Fig. 4A and Supplementary Material 4). The members of GATA family were consisted of GATA1 to GATA6 and the atypical protein TRPS1. GATA1/2/3 was necessary for the differentiation of mesoderm and ectoderm, such as the nervous and hematopoiesis systems, GATA4/5/6 was essential for the development of mesoderm and endoderm, including cardiovascular embryogenesis [36, 37], and TRPS1 was reported to modulate a number of major cellular processes [38–41], such as tumor cell proliferation and apoptosis [42–44]. The associations among PR and these proteins were explored by TCGA data bank, the data demonstrated that there was positive correlation between PR and TRPS1 in endometrial cancer (*R* = 0.42) and the coefficient of PR and TRPS1 was the highest in breast cancer (*R* = 0.31), implying that there might exist potential interactions between PR and TRPS1 (Fig. 4B and Supplementary Fig. 6).

The GATA-type zinc finger transcription factor TRPS1 could function as a novel context-dependent tumor suppressor [44, 45]. According to the studies of researchers, the interaction between PR and the known steroid hormone receptor-associated co-factor TRPS1 could be induced by progesterone treatment [46], and TRPS1 could function as a coregulator recruitment to the PR complex [47, 48]. Thus, this protein was chosen for further analysis. From GEO datasets, TRPS1 expression was significantly lower in endometrial cancer progressive specimen compared with endometrial cancer non-progressive specimen in GSE29436, while the TRPS1 level was markedly higher in human breast cancer metastatic cell model than in normal breast cell line based on GSE107209 (Fig. 4C). The gene expression profile of TRPS1 across all tumor samples and paired normal tissues was detected in GEPIA database. As shown in Fig. 4D, the expression of TRPS1 was significantly elevated in BC tissues compared with matched normal tissues, and was decreased in EC tissues but the difference was not significant. Meanwhile, we found that promoter methylation of TRPS1 in BC was dramatically down-regulated in comparison to normal breast tissues, while no remarkable DNA methylation difference was detected in EC tissues and normal endometrial tissues (Fig. 4E), suggesting that TRPS1 expression may be attributed to DNA methylation. Immunohistochemical staining indicated that the expression levels of TRPS1 were significantly lower in endometrial tumors than those in normal tissues, whereas TRPS1 was highly expressed in BC tissues compared to normal tissues (Fig. 4F).

Based on the previous studies, TRPS1 executed transcription repression function through interacting with multiple components of the nucleosome remodeling deacetylase (NuRD) complex, such as HDAC2 to establish the precision-guided machinery [42], loss of TRPS1 expression reduced DNA binding potential of HDAC1 and HDAC2 [44], thus regulating histone acetylation and deacetylation of the target genes [49]. According to the analysis of GEPIA, the findings indicated that PR positively correlated with TRPS1 (*R* = 0.42) and TRPS1 positively correlated with HDAC2 (*R* = 0.44) in EC, while in breast cancers, the expressions of PR and TRPS1 were positively correlated (*R* = 0.31), the association between TRPS1 and HDAC2 was not significant (*R* = 0.058), which may be owing to different cell contexts (Fig. 4B, G). To further verify the interactions between PR and TRPS1 complex, exogenous Co-IP assays were performed in 293 T cells, which displayed that PR and TRPS1 showed a mutual interaction and TRPS1 could also bind with HDAC2 (Fig. 4H), implicating that there may exist PR/TRPS1/HDAC2 complex involving in transcription regulation, whereas the status of the complex in different cells warranted our additional investigation.

### The PR/TRPS1/HDAC2 complex was involved in the regulation of RANKL by MPA in cell lines

**Fig. 5:**
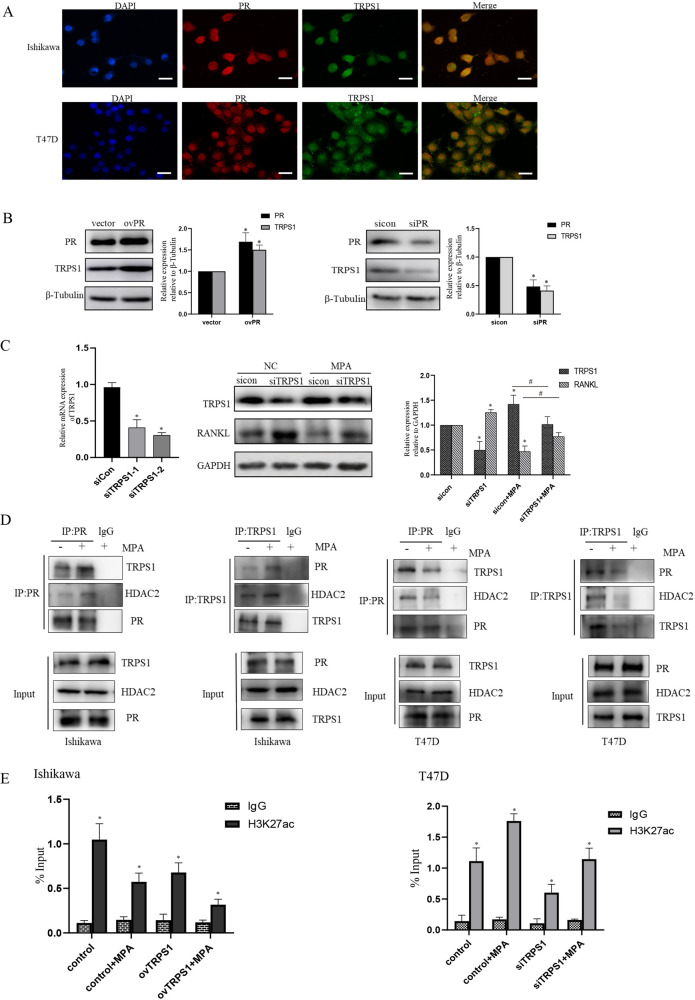
The recruitment of TRPS1 by PR to the promoter of RANKL modulated the expression of RANKL through histone acetylation. **A** The colocalization of PR and TRPS1 in Ishikawa and T47D cells detected by confocal microscopy. Cells were immunostained with anti-PR (red) and anti-TRPS1 (green) antibodies, DAPI (blue) was used to indicate cell nuclei. **B** The protein level of TRPS1 was analyzed after PR knockdown or overexpression by western blot. **C** RT-PCR assay was used to determine TRPS1 mRNA level after transfection with two independent siRNAs targeting TRPS1, then western blot assay was to validate its effect on RANKL expression with or without MPA. **D** The interrelationship of PR, TRPS1 and HDAC2 was verified in tumor cells after MPA treatment by endogenous IP experiments in Ishikawa and T47D cells, respectively. **E** ChIP-qPCR assay was to examine the status of H3K27Ac enrichment at distal PRBS of RANKL after indicated treatment in EC and BC tissues, respectively. *p < 0.05, compared with control group. ^#^*p* < 0.05, compared with indicated groups.

As PR was found to interact with TRPS1 complex, experiments were conducted to further validate their reciprocal effect. From immunofluorescence staining, we discovered that endogenous PR and TRPS1 were co-localized in the nuclei of both EC and BC cells (Fig. 5A). Furthermore, transfection of PR plasmid resulted in the enhanced expression of TRPS1, knockdown of PR synchronously suppressed the profile of TRPS1, suggesting that the expression of TRPS1 exhibited changes occurring in parallel with alterations in PR expression (Fig. 5B). Next, FISH tests demonstrated that no case was positive for TRPS1 and PR fusions (Supplementary Fig. 7). Furthermore, silencing of TRPS1 by TRPS1-targeted siRNAs led to a decrease in its mRNA and protein. Meanwhile, the attenuated RANKL expression induced by MPA was alleviated by TRPS1 abolishing in EC cells (Fig. 5C). In addition, we explored the interactions of the PR/TRPS1/HDAC2 complex after MPA treatment in cancer cell lines. As shown in Fig. 5D, the utilization of MPA enhanced the interactions between PR, TRPS1 and HDAC2 in Ishikawa, while the binding capacity of PR, TRPS1 and HDAC2 was remarkably abrogated in the presence of MPA in T47D cells.

As HDAC2 was an essential deacetylase to modulate histone acetylation and deacetylation level of target genes, we hypothesized that interfering with the component of the PR/TRPS1/HDAC2 complex may affect the acetylation level of its downstream gene RANKL. ChIP-qPCR of histone modifications revealed that H3K27Ac, an active histone mark, was less enriched in the distal PR binding sites of RANKL gene in TRPS1 overexpression group versus control group, and MPA treatment further reduced this enrichment in Ishikawa. While in T47D cells, MPA increased H3K27Ac binding to the distal enhancer, TRPS1 silencing abrogated this MPA-mediated effect (Fig. 5E). These histone modification patterns not only supported our observation of MPA-regulated RANKL transcription in EC and BC cells, but also indicated a more accessible chromatin structure adjacent to the RANKL gene in cancer cells.

### The alterations of TRPS1 affected progestin sensitivity

**Fig. 6:**
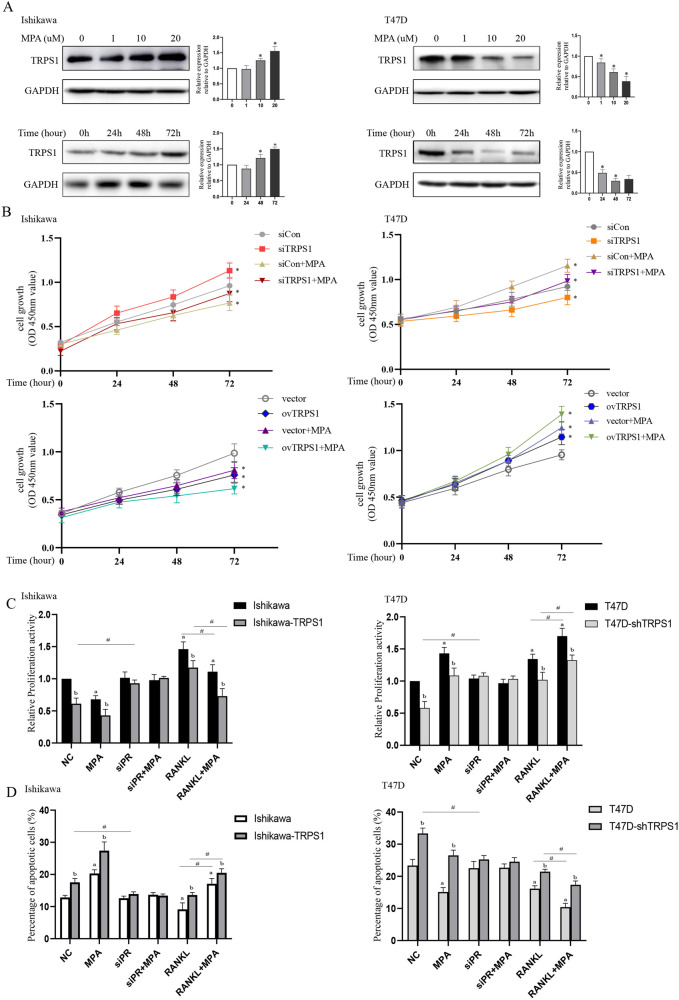
TRPS1 participated in regulating the function of MPA on endometrial cancer cells and breast cancer cells. **A** MPA regulated the protein level of TRPS1 in a dose- and time-dependent manner in Ishikawa and T47D cells, respectively. **B** Knockdown or overexpression of TRPS1 affected the effect of MPA on tumor cells of endometrium and mammary glands using CCK8 methods. **C** Cell proliferation assay was employed to assess the role of TRPS1 in indicated treatment after constructing TRPS1 overexpression or knockdown stable cell lines. **D** Cell apoptosis was evaluated by flow cytometry in stably transfected cell line with overexpression or knockdown of TRPS1 after relevant treatment. *p < 0.05. ^a^Compared with negative control; ^b^compared with its counterpart group; ^#^compared with indicated groups.

Recent studies pointed out that TRPS1 had a critical role in maintaining epithelial cell growth [44, 50], but whether it was involved in mediating the function of MPA remained ambiguous. We firstly treated endometrial cancer cells and mammary cancer cells with different doses of MPA, and tested its effects on TRPS1 at multiple time points. The results showed that MPA enhanced TRPS1 expression in a time- and dose-dependent manner in EC cells, while repressed its expression in BC cells (Fig. 6A and Supplementary Fig. 8), which was consistent with its regulation at the transcriptional level (Supplementary Fig. 9). In Ishikawa, Loss of TRPS1 significantly prompted the growth of endometrial cancer, and impaired the inhibitory effect of MPA on it. In addition, overexpression of TRPS1 reinforced the efficacy of progestin in suppressing the proliferation of EC cells. On the contrary, depletion of TRPS1 inhibited cell proliferation induced by MPA treatment in T47D cells and transfecting with the TRPS1 plasmid accelerated progesterone-driven breast cancer cell growth (Fig. 6B).

Meanwhile, Ishikawa cells were transfected with TRPS1 plasmid to generate the stable cell lines (Ishikawa-TRPS1) by the lentiviral packaging system. Ishikawa-TRPS1 cells were more susceptible to MPA treatment than Ishikawa vector control cells, and interfering with the expression of PR blocked the effect of MPA on cell growth in both Ishikawa and Ishikawa-TRPS1 cells. The addition of RANKL promoted cell viability more significantly in Ishikawa than that in Ishikawa-TRPS1, while MPA treatment impaired the cancer-promoting effect of RANKL on both cells. Additionally, T47D cells were transfected with TRPS1 shRNA to obtain stable knockdown of TRPS1 (T47D-shTRPS1) by transducing lentiviral particles. T47D cells with TRPS1 silencing presented a lower cell growth rate than the control group, and loss of PR blocked this suppression. MPA-induced cell proliferation was inhibited in T47D-shTRPS1 cells. The utilization of RANKL facilitated MPA-mediated cell viability, while the rate was lower in T47D-shTRPS1 cells than in its counterpart group (Fig. 6C).

Subsequently, flow cytometry was applied to determine the role of TRPS1 in cell apoptosis. The Ishikawa cells with TRPS1 overexpression exhibited a significantly higher rate of apoptosis than the control, and the effect was enhanced in the presence of MPA, but was remarkably blocked by transfection with siPR. Meantime, treatment with RANKL suppressed MPA-induced cell apoptosis in Ishikawa-TRPS1 group. While T47D cells with TRPS1 depletion showed a higher apoptotic rate than the control and weakened the antiapoptotic effect of MPA, but this effect was disappeared when transfection with siPR. The combination of MPA and RANKL significantly inhibited tumor apoptosis, but knockdown of TRPS1 suppressed this effect (Fig. 6D). These data suggested that TRPS1 was involved in the critical function of MPA/PR on cell proliferation and apoptosis.

### TRPS1 controlled the impact of MPA on tumor growth in murine xenograft model

**Fig. 7:**
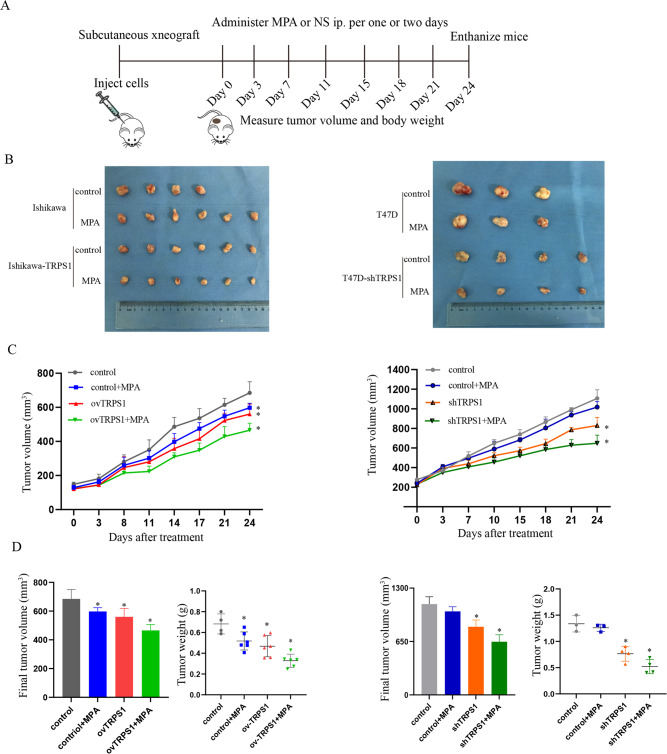
Involvement of TRPS1 in MPA-controlled cell growth in vivo. **A** The flowcharts of subcutaneous xenograft and drug treatments for nude mice. **B** Representative images of xenograft tumors subjected to the indicated treatments. **C** Tumor volumes were shown. The data were present in mean ± standard deviation. **D** The final volumes and weights of the xenograft tumors isolated from mice. *p < 0.05.

To determine the potential function of TRPS1 in vivo, we made use of orthotopic tumor model by injecting Ishikawa, Ishikawa-TRPS1, T47D, T47D-shTRPS1 stable cell lines into nude mice, respectively, and tumor growth was monitored over time. The process of subcutaneous xenograft and treatment schedule for in vivo study was shown in Fig. 7A. In addition, at the end of treatment, the mice were sacrificed and then their tumor tissues were collected for photographing (Fig. 7B). We could also see from the tumor growth curve and found that in the Ishikawa-TRPS1 group, tumor growth in nude mice was significantly suppressed after MPA treatment. Likewise, in the T47D-shTRPS1 group, TRPS1 silencing exerted a much stronger inhibitory effect upon tumor growth compared with control group (Fig. 7C, D). This finding was line with the expression patterns of cofactor TRPS1 that it could act as both an activator and a repressor of transcription which appeared to be context dependent.

## Discussion

Medroxyprogesterone (MPA) played an anticancer role in endometrial cancer, while increased the incidence of breast cancer according to multiple clinical studies [12]. Based on our previous researches, MPA attenuated the oncogenic behavior of RANKL in EC [18], while it promoted breast tumorigenesis by activating RANKL [51]. Shang, Y. et al., reported that tamoxifen induced the recruitment of coactivators of ER, facilitating the incidence of EC, but stimulated the recruitment of corepressors to prevent breast cancer [22, 23]. To this end, we aimed to explain whether PR could also recruit cofactors to modulate the selective mechanism of MPA in EC and BC.

**Fig. 8:**
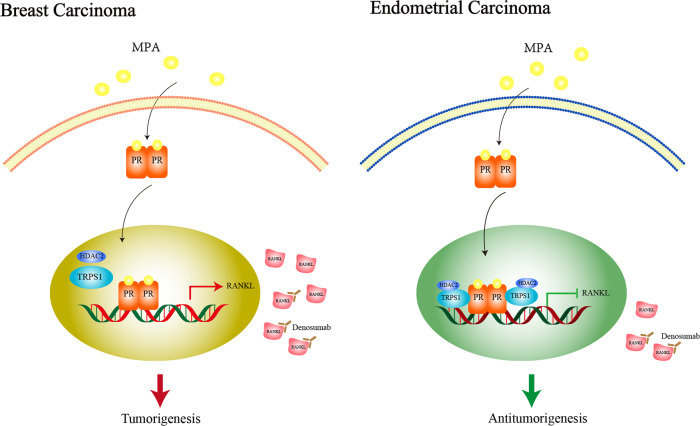
Proposed model for the potential function of TRPS1 on the selective mechanism of MPA in EC and BC cells. TRPS1 functioned as a cosuppressor recruited by PR combining with HDAC2 to enhance the sensitivity of cells to MPA via deacetylating RANKL in EC cells, while in different cellular contexts of breast cancer, TRPS1 served as a coactivator and the complex of PR/TRPS1/HDAC2 was disassociated, resulting in the acetylation of RANKL and facilitating the cancer-promoting effect of MPA in BC. Denosumab, as a monoclonal antibody directed to RANKL, could effectively enhance the tumor suppressor effect of MPA in EC, while inhibiting the growth of breast cancer.

In this current study, we found that TRPS1 functioned as a novel context-depended transcription coregulator recruited by PR. In EC, upon MPA treatment, TRPS1 could act as a transcription corepressor and interact with HDAC2 to form a PR/TRPS1/HDAC2 complex to deacetylate RANKL, downregulating the expression of RANKL and thereby exerting an anti-tumor effect. However, in BC, after MPA treatment, TRPS1 was identified as a transcription coactivator, PR recruited TRPS1 but did not interact with HDAC2 in the specific breast cell context, thus accelerating the acetylation of RANKL and enhancing the cancer-promoting effect of MPA (Fig. 8). Our findings provided a theoretical foundation for the tissue selection effect of MPA in endometrium and breast, and demonstrated that the context-dependent effect of TRPS1 played a crucial role and could be utilized as a therapeutic target for the treatment of endometrial cancer while inhibiting the occurrence of breast cancer.

The anti-proliferative action of progestogens was observed in the endometrium which might be just opposite in the breast [52–54]. The molecular mechanism underlying this ambivalent phenomenon of MPA had remained elusive. Numerous studies in recent years had contributed pivotal data on the role of progestogens and the involvement of RANKL, which was initially discovered within the immune and the bone systems, and gained renewed attention over the past decade as a hot topic in cancer research [55, 56]. In our previous study, we detected that higher RANKL expressions were explored in endometrial carcinomas with more aggressive clinical features, MPA could block the migratory and invasive capacities of EC cells induced by RANKL via PR [20]. Schramek et al. reported that in vivo administration of MPA triggered massive induction of the key osteoclast differentiation factor RANKL in mammary-gland epithelial cells, markedly increasing the risk of developing breast cancer [21]. Consistent with these researches, our current study further illustrated that MPA repressed the expression of RANKL in a dose- and time- dependent manner. RANKL knockdown significantly impaired cell growth and enhanced the inhibitory effect of MPA on cell apoptosis in EC cells. While in BC cells, MPA elevated RANKL expression, silencing RANKL resulted in the suppressive proliferative and apoptotic capacity mediated by MPA. These interesting findings suggested that there existed tissue selective effect of MPA on the expression of RANKL and RANKL controlled the MPA-mediated cell viability and apoptosis of endometrial and mammary cancer.

Progestins exerted their complex and context-dependent effects by binding to PR and then cross-talked with transcriptional coactivators and corepressors, including TRPS1 [34, 57]. The transcription factor TRPS1 with classic GATA-type zinc fingers, was known to repress transcription [58, 59], prior studies had also hinted at roles for overexpressed TRPS1 in tumorigenesis, including breast cancer [60]. Witwicki et al. pointed out that TRPS1 might act as both an activator and a repressor of transcription in specific tumor environment [42]. In line with this, we found that in EC, PR could recruit the cofactor TRPS1 and combine with HDAC2 forming a PR/TRPS1/HDAC2 corepressor complex to the chromatin of RANKL, decrease the H3K27Ac level of RANKL, repress RANKL transcription, thus reinforcing MPA-mediated anti-tumorigenic effect. Whereas in the context of BC cells, MPA treatment enhanced the interaction between PR and TRPS1, but had no effect on the interaction between TRPS1 and HDAC2, so it induced the enrichment of H3K27Ac at the RANKL chromatin and elevated the transcription of RANKL, thereby facilitating BC development. These modulation patterns were in agreement with the points of Davaadelger et al. that TRPS1 was identified as a coregulator recruited to PR sites and could serve as a PR corepressor [47]. Subsequently, Elster and Wang suggested that TRPS1 could recruit corepressor complexes to chromatin [61, 62]. Our findings further revealed that in EC, PR recruited TRPS1/HDAC2 forming a transcriptionally repressive, precision-guided machinery to the chromatin and suppressed the expression of target gene RANKL under MPA treatment, which coincided with the research of Cornelissen that TRPS1 could interact with the core enzymatic components of the NuRD complex (HDAC2) [44], thus regulating gene transcription by histone deacetylation [63]. On the other hand, our researches about MPA-driven breast carcinogenesis through PR/TRPS1-mediated acetylation were supported by the studies that TRPS1 might emerge as a transcription activator and induce the expression of its downstream genes [43, 64, 65].

Moreover, we also verified the complicated role of TRPS1 in tumor xenograft models and found that TRPS1 overexpression resulted in a potent decrease of cell growth when coupled with MPA in endometrial cancer, and loss of TRPS1 expression in BC cells showed reduced MPA-mediated growth pattern. Likewise, Cornelissen made a similar point that several cancer cell lines showed reduced growth both in vitro and in vivo upon TRPS1 knockdown [42, 61, 66], and in other settings, silencing TRPS1 seemed to be promoting tumor growth [44], further implying that TRPS1 expression was essential for proliferation and the effects of TRPS1 appeared to be context dependent. Based on the modulation of RANKL via the recruitment of coregulator TRPS1 by PR, we also made use of the denosumab, a fully human IgG2 monoclonal antibody that bound human RANKL and blocked it from oligomerizing its receptor, thereby suppressing the effect of RANKL [28, 67]. In both EC and BC cells, we observed that denosumab could execute inhibitory effects on tumor growth, especially in EC cells when combined with MPA treatment. However, its extensive clinical applications in endometrial cancer, particularly in progesterone-insensitive endometrial cancer still needed further exploration.

In summary, the recruitment of cofactor TRPS1 by the activated PR differently altered the acetylation level of RANKL via establishing the PR/TRPS1/HDAC2 complex in EC and BC, thus affecting tumor behaviors. Our study provided novel and important insights into the crucial role of coregulator TRPS1 and presented rationale for further elucidation of PR/TRPS1-mediated transcriptional regulation in the selective mechanism of MPA from a genetic and epigenetic perspective. Dissecting the relationship between PR cofactor recruitment and histone modification will improve our understanding of the complex selective mechanisms of progesterone and assist us to identify TRPS1 as a potential marker for personalized therapeutic strategies in endometrial carcinoma.

## Materials and methods

### Cell cultures and transfection

The human endometrial cancer cell line Ishikawa and HEC-1A, breast cancer cell line T47D, MCF7 and human embryonic kidney (HEK) 293 T cells were purchased from American Type Culture Collection (ATCC) without mycoplasma contamination. Specifically, we cultured Ishikawa cells in DMEM/F12 medium (Gibco), HEC-1A cells in McCOY’s 5 A medium (Sigma), T47D cells (PR+/ER+/Her2−) in RPMI1640 medium (Gibco), MCF7 (PR+/ER+/Her2−) and HEK-293T cells in DMEM medium (Sigma). All culture media contained 10% fetal bovine serum (FBS, Gibco), 100 U/ml penicillin G and 100 µg/ml streptomycin (Life Technologies) and all cell lines were cultured at 37 °C in a humidified chamber with 5% CO_2_.

The lentivirus for TRPS1 knockdown (shTRPS1) and TRPS1 overexpression (ovTRPS1) were obtained from GenePharma (Shanghai, China). For stable transduction, Ishikawa cells and T47D cells were transfected with lentivirus for TRPS1 knockdown and overexpression based on the manufacturer’s protocol [68]. Next, we used the puromycin to select stable cells. The other siRNAs and plasmids were synthesized by GeneChem (Shanghai, China). Cell transfections were carried out using Lipofectamine 3000 reagent (Thermo Scientific) with Opti-MEM reduced serum medium (Thermo Scientific). The sequences of shTRPS1 and siRNAs were shown in Supplementary Table 1.

### Co-immunoprecipitation (Co-IP) and western blotting analysis (WB)

Exogenous and endogenous co-IP assays were performed as described previously [69]. After cell transfection, cells were lysed in the relevant HEGN buffer (Thermo Scientific, 26146) containing protease inhibitor cocktail (Sigma), then cleared protein lysates were incubated with antibody-coupled Dyna beads (Thermo Scientific) overnight at 4 °C. The immunoprecipitates were washed with HEGN buffer, then boiled with sample buffer and subjected to immunoblotting analysis. Primary antibodies were as follows: PR (Abcam, ab206926), TRPS1 (Abcam, ab125197), HDAC2 (Abcam, ab32117), lgG (Abcam, ab133470), HA-tag (MBL, M180-7), His-tag (MBL, CW0285M), Flag-tag (MBL, M185-7).

For western blotting assay, total protein was lysed and extracted by RIPA buffer. After measuring protein concentration, 50 µg protein was loaded to SDS-polyacrylamide gel, electrophoresed and transferred to the PVDF membranes, following incubated overnight with primary antibodies against PR (Cell Signaling Technology, 8757), RANKL (Cell Signaling Technology, 4816), TRPS1(Cell Signaling Technology, 17936), GAPDH (Abcam, ab8245), β-Tubulin (Abcam, ab6046), respectively. After incubating with the indicated secondary antibodies, detection was carried out using the chemiluminescence detection system (ECL detection kit, Millipore). Each experiment was performed for at least three times.

### RNA extraction and quantitative real time PCR analysis (qRT-PCR)

RNA isolation and qRT-PCR analysis were performed as previously presented [70]. Briefly, total RNA was extracted with Trizol reagent (Sigma). Two µg of total RNA was reverse transcribed into cDNA according to the standard procedures of the reverse transcriptase kit (TransGen, AE341-02). PCR reaction was performed in technical triplicates using PCR SuperMix (TransGen, AS111-01). The expression values were analyzed with ABI 7300 detection system (Thermo Scientific). The used PCR primers are listed in Supplementary Table 2.

### Immunohistochemistry staining (IHC)

Paraffin-embedded 95 samples (including 25 cases of proliferative phase and 70 cases of endometrial endometrioid cancer) were collected from the International Peace Maternity and Child Health Hospital (Shanghai, China) between Jan 2019 and Jan 2020. 12 cases of normal breast tissues and 36 cases of breast cancer tissues were constructed in a tissue microarray obtained from Shanghai Outdo Biotech Co., Ltd. (Shanghai, China). Pathological diagnosis was independently confirmed by at least two experienced pathologists. None of the patients had previously received hormonotherapy, adjuvant radiotherapy or chemotherapy before surgery. Our studies were conducted in accordance with the principles of the World Medical Association Declaration of Helsinki and ethical guidelines. Informed consent was obtained from each subject after approval by the hospital ethics committee. Five-micron slices for IHC staining were cut and placed on positively charged glass slides. Primary antibodies utilized in this assay included RANKL (Abcam, ab9957), TRPS1 (Zenbio, R50197). The analysis of RANKL and TRPS1 protein expression levels was performed as previously described [71, 72] and the results were assessed by two independent observers using a semi-quantitative method without acknowledging the clinical pathological parameters.

### Cell proliferation and apoptosis analysis

The viability of cells was tested by Cell Counting Kit‑8 (CCK‑8, Japan). Cells were seed in 96-well plate in triplicate for about 2000 cells/well. After cell treatment for the indicated time, the detection solution was added to the wells and incubated for about 4 h. The absorbance was tested at 490 nm in the end. All the operation steps were in strict accordance with the protocols and the assay was performed in independent triplicates [73].

The cell lines were seeded in 6-well plates with a density of 5 × 10^5^/ml. After treatment and incubation for the appropriate time, cells were collected, washed with ice-cold PBS, and resuspended with 1× binding buffer containing Annexin V-FITC and PI (BD, USA). After incubated in the dark for 15 min at room temperature, the samples were detected by flow cytometer (BD Biosciences).

### Immunofluorescence and fluorescence in situ hybridization (FISH) assay

Cell immunofluorescent staining was performed as previously presented [74]. The cells were fixed, permeabilized and blocked, then primary antibodies against PR (Santa Cruz, sc-810) and TRPS1 (Sigma Aldrich, HPA060380) were incubated with cells overnight at 4 °C, respectively, followed by incubation with secondary fluorescent-tagged antibody (Alexa Fluor® 488 and 555, Life Technology) for 1 h at room temperature (RT). Nuclei were counter-stained with 4′, 6-diamidino-2-phenylindole (DAPI) (Beyotime, China) for 5 min. Fluorescence was captured using a fluorescence microscope (Nikon).

In order to visualize the distribution and expression of PR and TRPS1, FISH assays were carried out [75]. Cells were fixed, permeabilized and prehybridized. Then we performed the hybridization with probes in the dark at 37 °C overnight. Next, we rinsed cells at 42 °C in SSC buffer. The PBST containing 5% BSA was utilized as the blocking buffer. Subsequently, we incubated the cells with primary antibody at RT for 1 h. Cells were incubated with secondary antibodies and DAPI. The confocal microscope was utilized to capture the images. The sequences are presented in Supplementary Table 3.

### Dual-luciferase reporter assays

Cells were transfected with pGL4.10-RANKL promoter plasmid, firefly plasmid, and pGL4.74 [hRluc/TK] renilla plasmid, which acted as the internal control. The pGL4.10-RANKL promoter plasmids contained 2000 bp upstream of the transcription initiation site of RANKL. The primers are listed in Supplementary Table 4. The wild-type (WT) plasmid of pGL4.10-RANKL involving the two putative PR-binding sites (PRBS) (GGATGTT; AACATAT), or the mutated (Mut1) plasmid involving the second PRBS (AACATAT) with point mutation at the first site, or the mutated (Mut2) plasmid involving the first PRBS (GGATGTT) with point mutation at the second site were co-transfected together with PR overexpression plasmid or its vector control plasmid using lipofectamine 3000 (Invitrogen) according to the manufacturer’s instructions [76]. Then the cells were lysed to detect the luciferase activity by VARIOSKAN FLASH (Thermo Scientific) using a dual luciferase reporter kit (Promega, E1910).

### RNA-Sequencing analysis

RNA-Sequencing experiments were performed in Ishikawa and T-47D cells. Cells were treated with MPA (20 mM) for 48 h. Then total RNA was extracted, RNA integrity was verified and library preparation was conducted with the NEBNext® Ultra RNA Library Prep Kit with Dual Index Primers. Cycles for amplification of the cDNA were determined using qRT-PCR. Then libraries were quantified with the Agilent 2100 Bioanalyzer electrophoresis system (Agilent Technologies) and subjected to Illumina Sequencing (HiSeq 2500) [46]. Experiment was performed in three replicates for each group.

### Gene Set Enrichment Analysis (GSEA)

Functional analysis of the RNA-Seq data were carried out using GSEA as follows [77]. All genes explored by RNA-Seq were ranked and weighted by their mean log2 fold change on progesterone treatments. These data were then analyzed using the GSEA v2.0.13 tool. The normalized enrichment score (NES) and FDR *q* value were applied to evaluate the enrichment effect of the gene set, and an FDR *q* < 0.05 was considered statistically significant.

### Chromatin Immunoprecipitation and ChIP sequencing

ChIP assay was performed based on the manufacture’s protocol [76]. Briefly, 2–3 × 10^7^ cells were crosslinked using 1% formaldehyde at room temperature for 10 min and then quenched by 125 mM glycine for 5 min. The fixed cells were resuspended in 1 ml of cell lysis buffer (1% SDS, 5 mM EDTA and 50 mM Tris-HCl, pH 8.1) containing protease inhibitors cocktail (Roche) and sonicated with Bioruptor® Plus sonication device (Bioruptor) to generate chromatin fragments. All experiments were performed in triplicate and normal IgG (Abcam, ab172730), 10 ug of PR (Cell Signaling Technology, 8757) and H3K27Ac (Abcam, ab4729) antibodies were used. Immunoprecipitated DNA was extracted with the QIAquick PCR purification kit (Qiagen, Cat. No. 28104) and DNA was subjected to real-time PCR analysis. Enrichment was calculated as a percentage of total input DNA. Primer sequences were listed in Supplementary Table 4.

ChIP sequencing (ChIP-seq) experiments were performed as previously described [61]. The Ishikawa cells were treated with control (DMSO) or MPA for 24 h. Genomic DNAs were prepared as described above. Then the purified DNA was subjected to sequencing library construction with Kapa Biosystems Hyper Prep kit (#KK8500) according to the manufacturer’s protocol. 1 ng of DNA was used, and 12-cycle library amplification was performed. Amplified libraries were checked on a Bioanalyzer (Agilent Technologies) and were sequenced on the IlluminaNextSeq500 with 75-bp single-end reads. Then the analysis was conducted as previously described [47].

### In vivo xenograft experiments

All of the animal experiments were conducted in strictly accordant with the Guideline for the Care and Use of Laboratory Animals, and approved by the department of Laboratory Animal Science at Shanghai Jiao Tong University School of Medicine. The BALB/c female athymic mice at 4 weeks of age were randomized divided into four groups (*n* = 6) and were injected with 1 × 10^7^ stably transfected cells resuspended in 100 µl of phosphate-buffered saline. Then, mice were intraperitoneally treated once a day with either DMSO (control) or MPA (20 mg/kg body weight). Tumor formation was closely monitored after injection and the volume was measured three to five days by digital calipers according to the formula Volume (mm^3^) = *L* (major axis) × W^2^ (minor axis)/2. The mice were sacrificed under anesthesia at 28 days after injection and the experiments were performed in an observer-blinded and randomized manner.

### Statistical analysis

All experiments data were obtained from at least three biological and technical repeats in this study. SPSS 19.0 (IBM SPSS Software) was applied for statistical analyses. Student’s *t* test, one-way or two-way ANOVA, and Spearman’s correlation analysis were utilized for further analyses. *p* values < 0.05 were considered as statistically significant when compared with control group. Error bars indicated standard deviation (SD) in the graphs.

## Supplementary information

Supplementary Figure1

Supplementary Figure2

Supplementary Figure3

Supplementary Figure4

Supplementary Figure5

Supplementary Figure6

Supplementary Figure7

Supplementary Figure8

Supplementary Figure9

Supplementary Figure legends

Supplementary material 1

Supplementary material 2

Supplementary material 3

Supplementary material 4

Supplementary Tables

Original Data File

## Data Availability

The datasets analyzed during the current study are available from the corresponding author on reasonable request.
