# Supplementary figures and images for: TRPS1 regulates the opposite effect of progesterone via RANKL in endometrial carcinoma and breast carcinoma
Source: Cell Death Discov. 2023 Jun 21;9:185. doi: 10.1038/s41420-023-01484-0 (PMC10284899; doi:10.1038/s41420-023-01484-0)

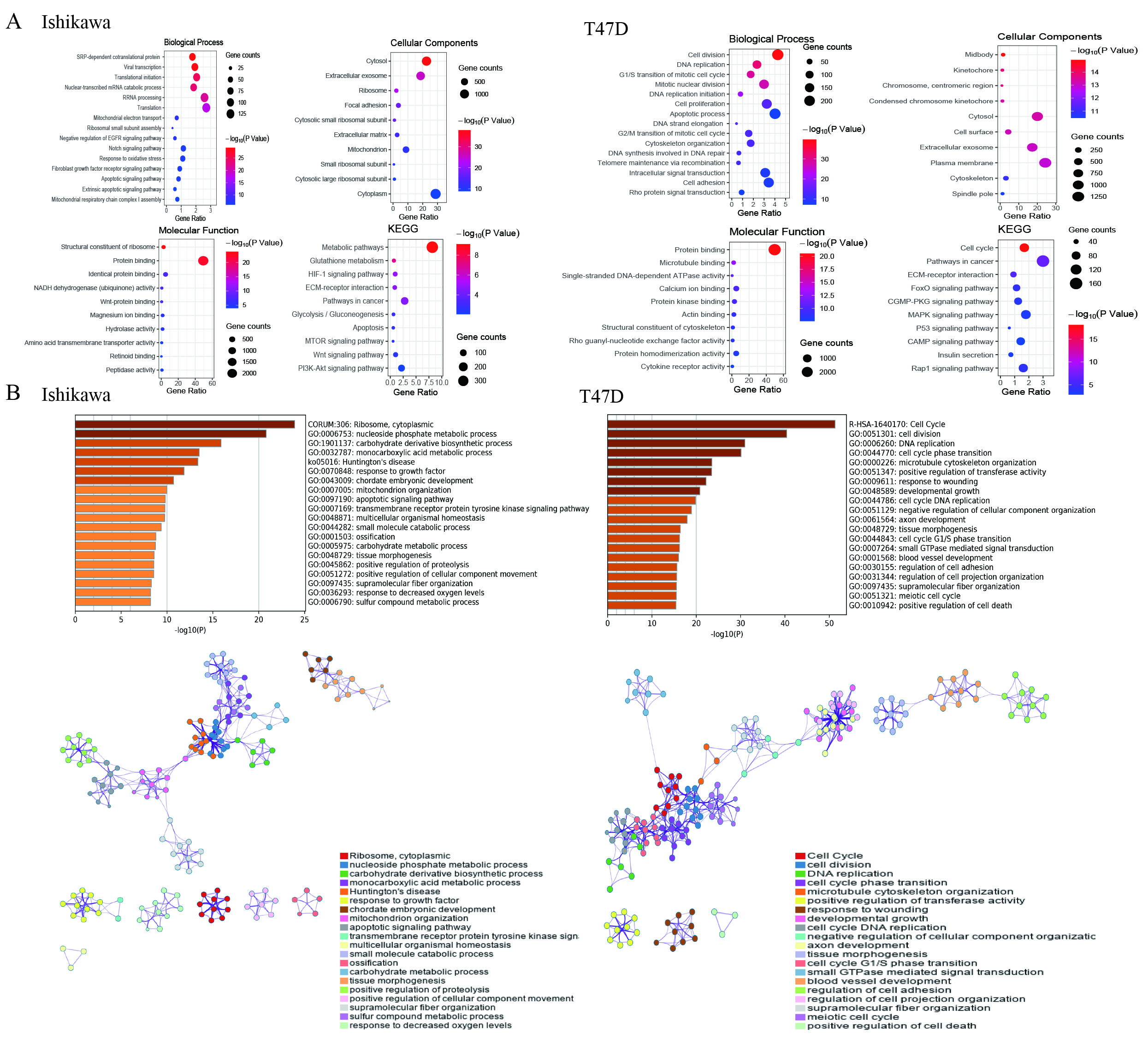

Supplement: Supplementary file 1 — Supplementary Figure1 [file 41420_2023_1484_MOESM1_ESM.jpg]

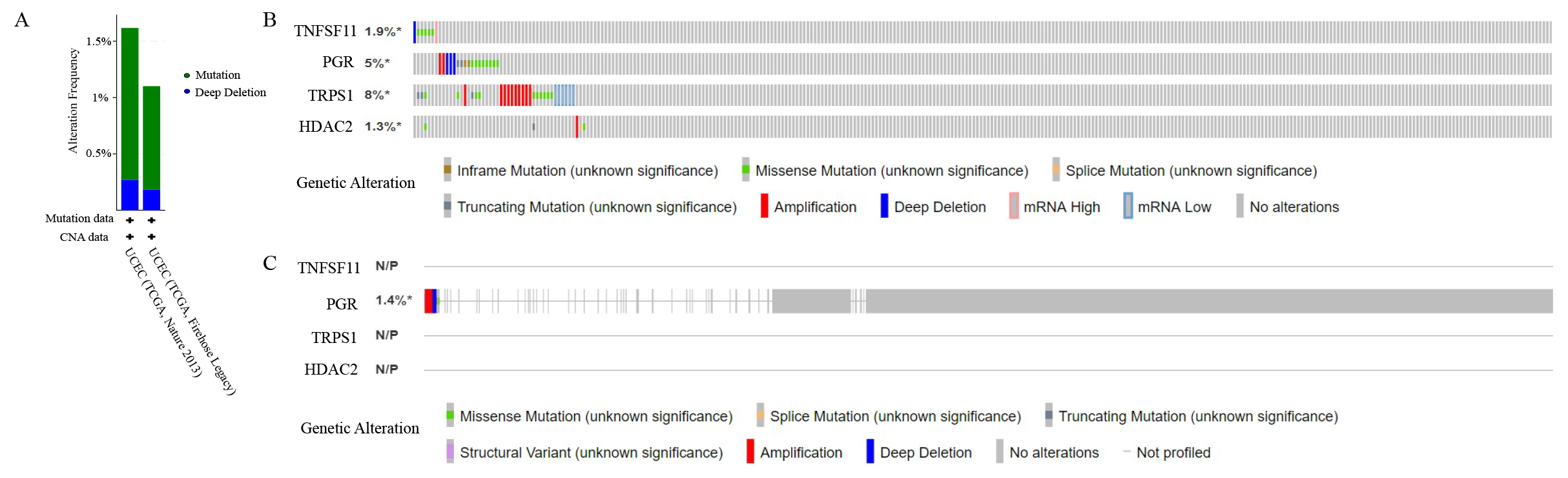

Supplement: Supplementary file 2 — Supplementary Figure2 [file 41420_2023_1484_MOESM2_ESM.tif]

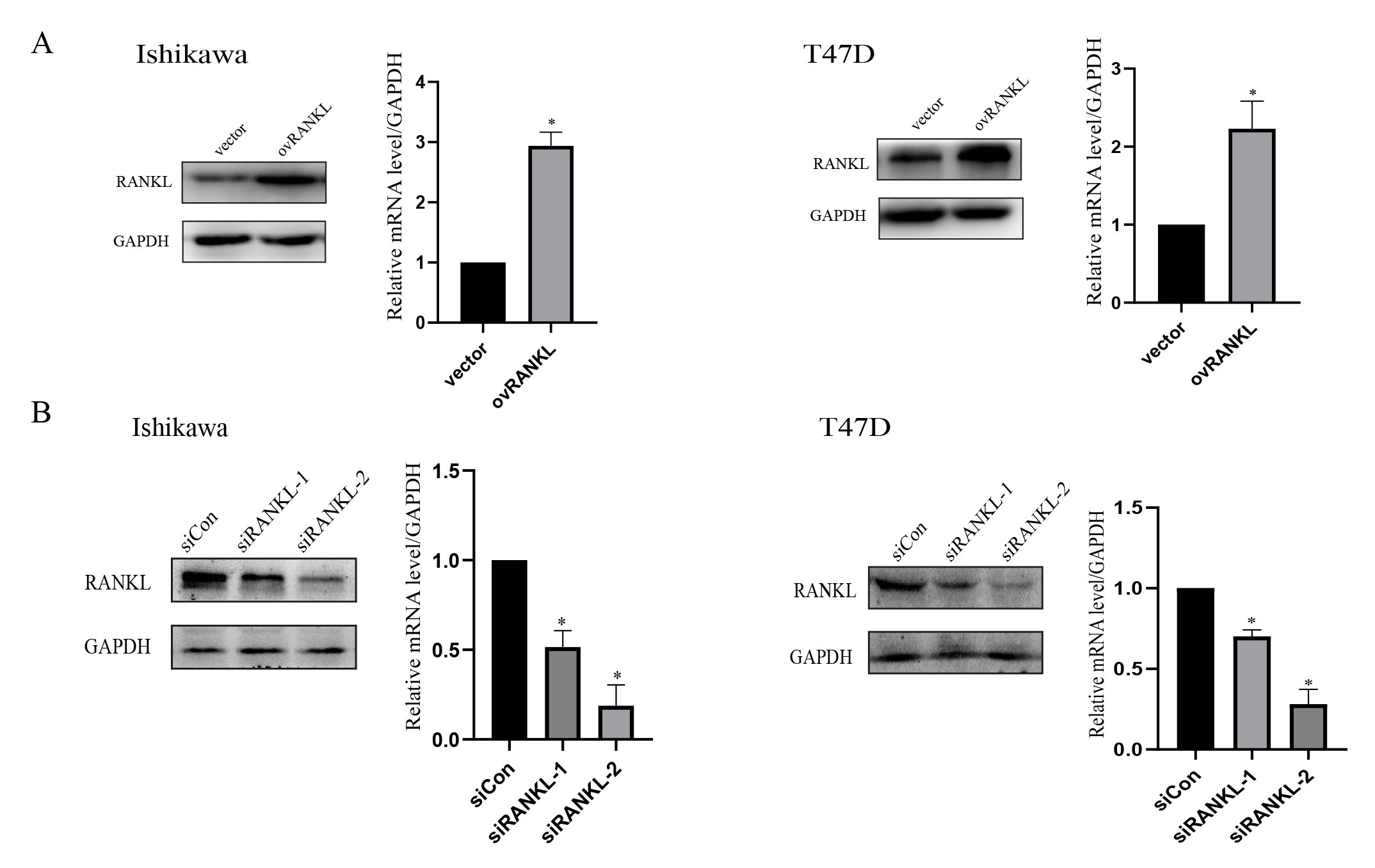

Supplement: Supplementary file 3 — Supplementary Figure3 [file 41420_2023_1484_MOESM3_ESM.jpg]

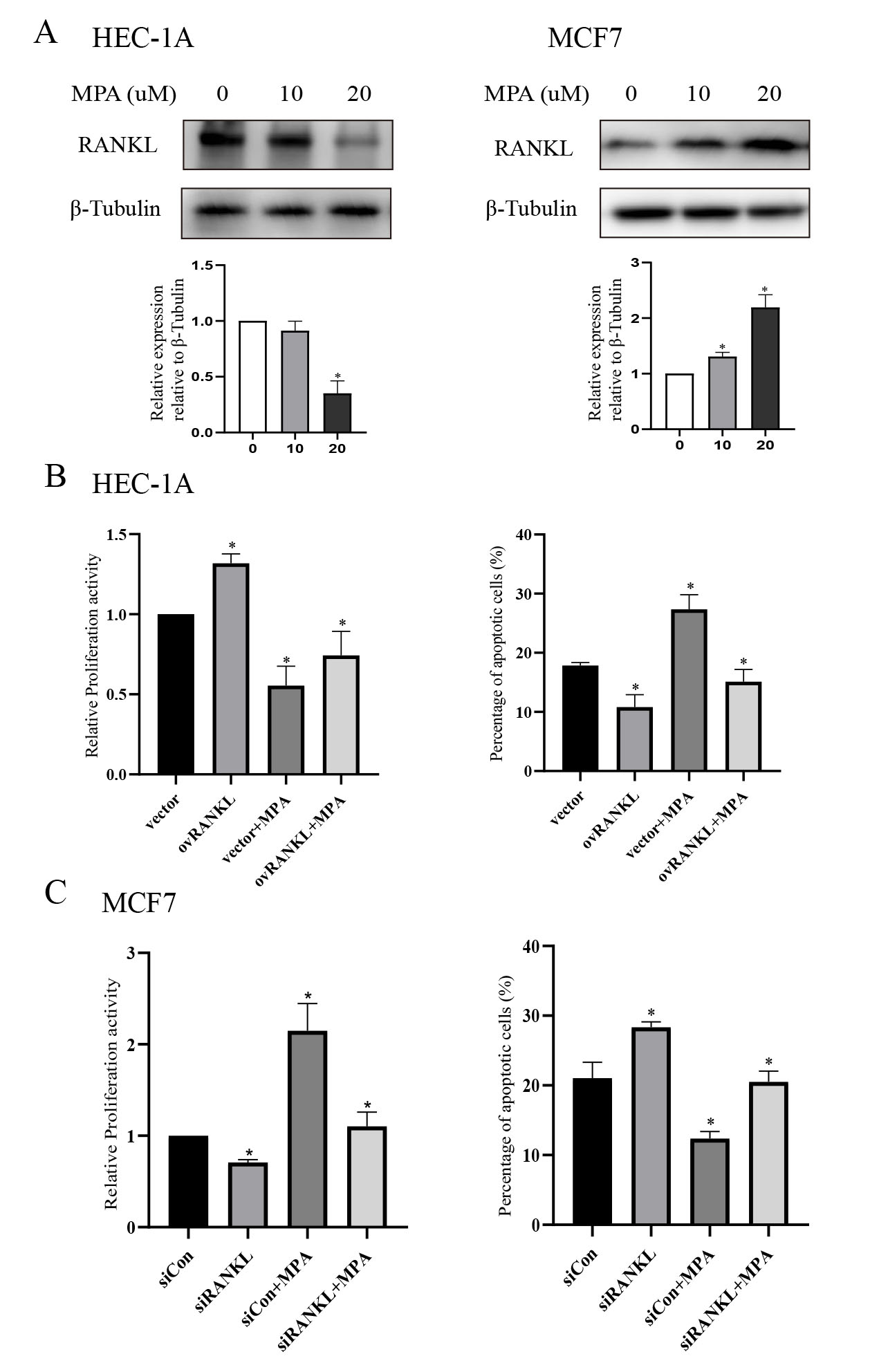

Supplement: Supplementary file 4 — Supplementary Figure4 [file 41420_2023_1484_MOESM4_ESM.jpg]

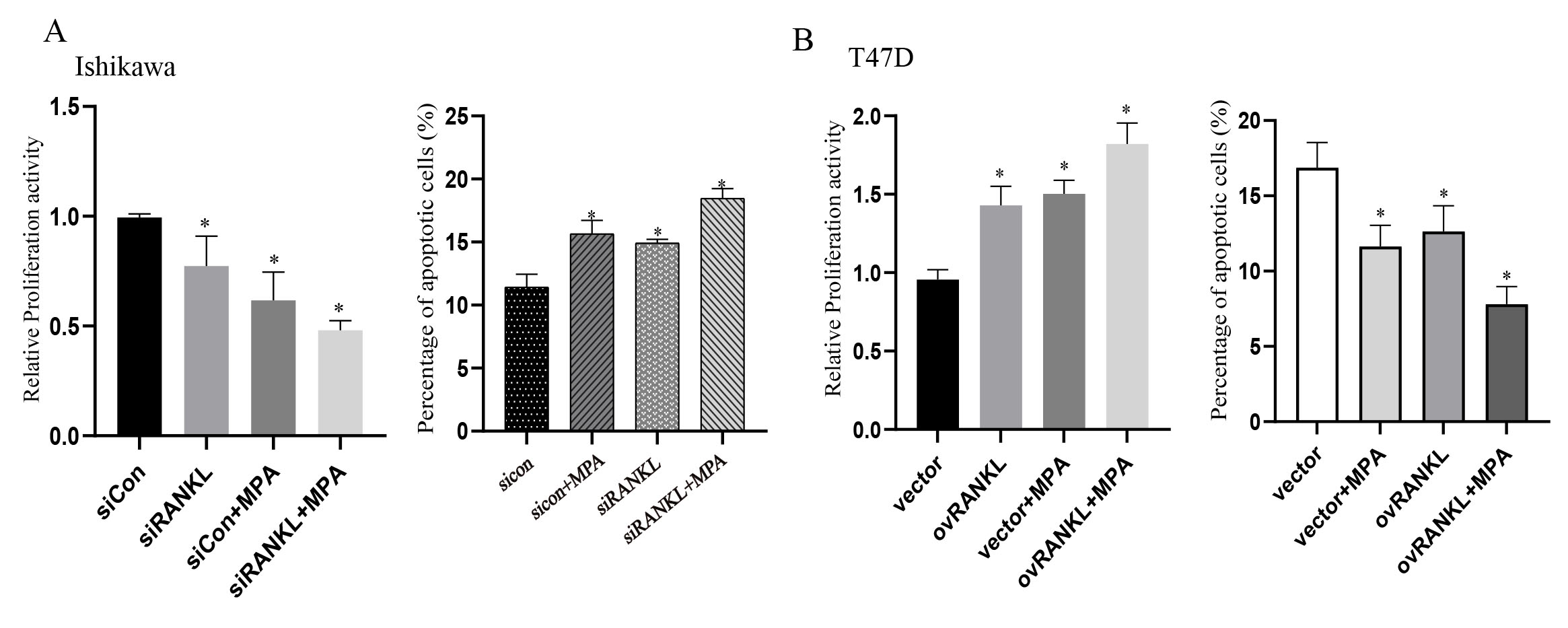

Supplement: Supplementary file 5 — Supplementary Figure5 [file 41420_2023_1484_MOESM5_ESM.jpg]

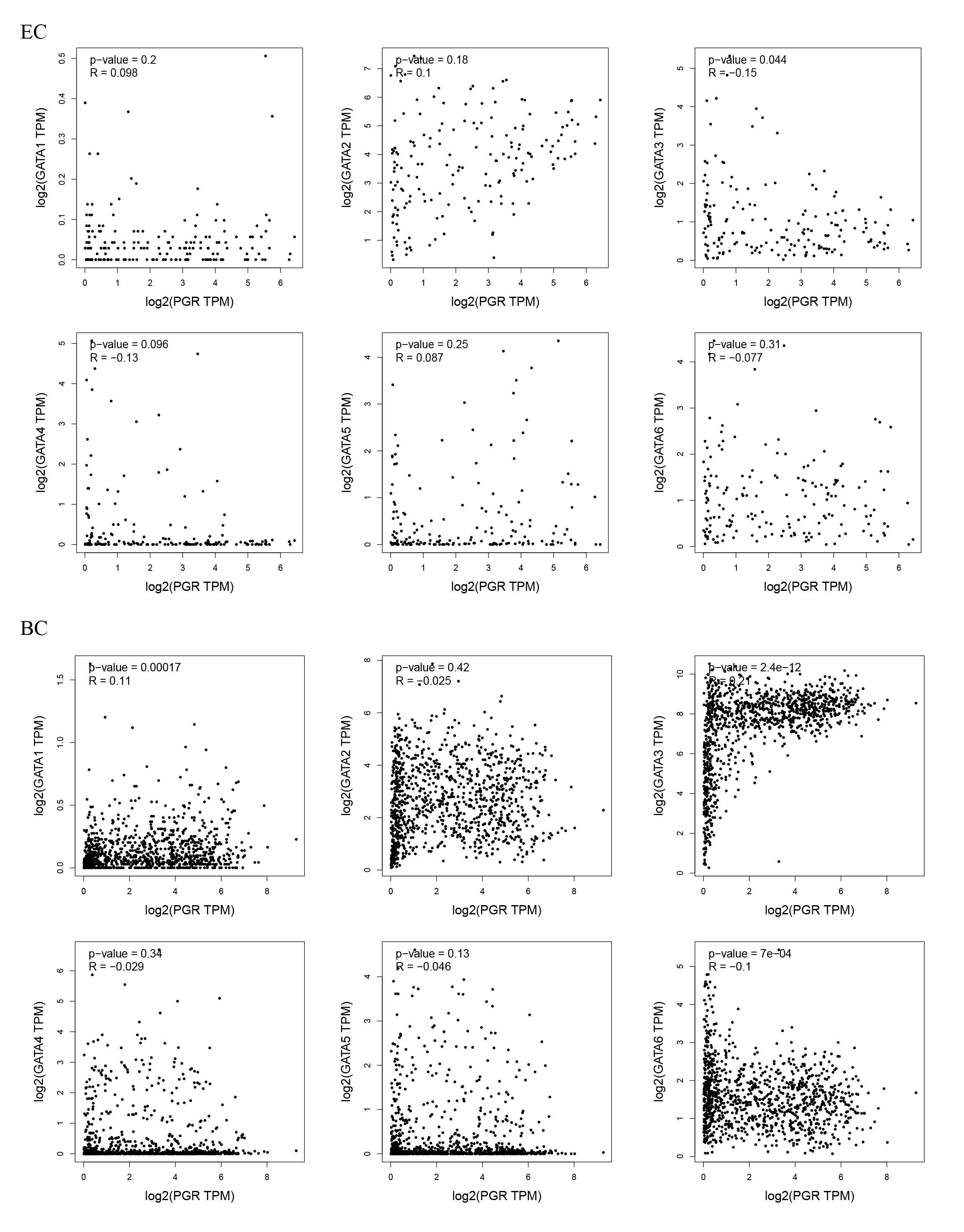

Supplement: Supplementary file 6 — Supplementary Figure6 [file 41420_2023_1484_MOESM6_ESM.tif]

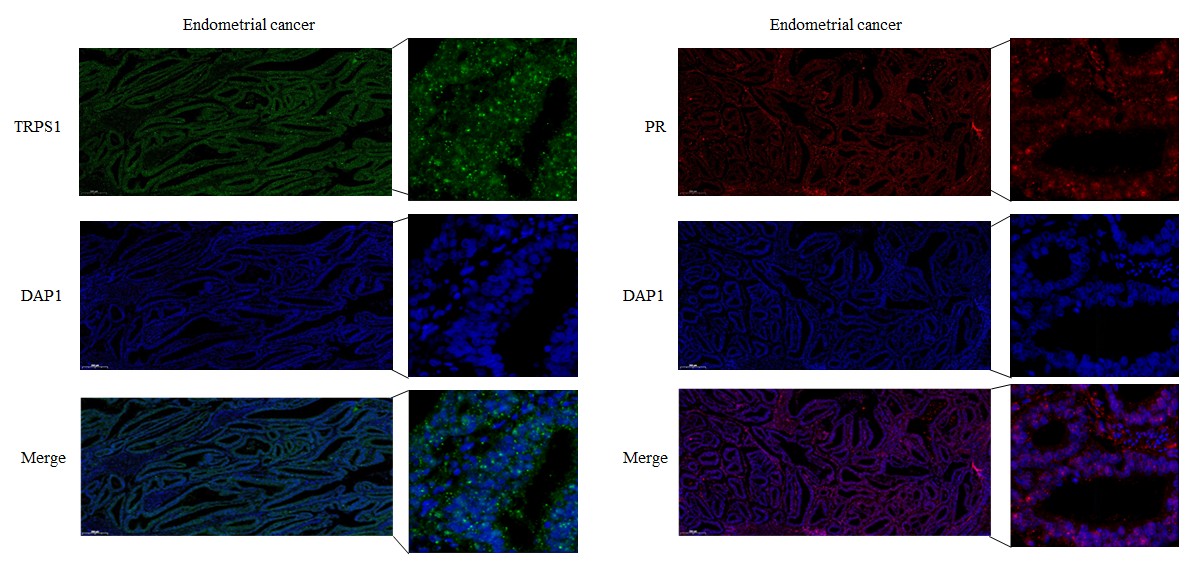

Supplement: Supplementary file 7 — Supplementary Figure7 [file 41420_2023_1484_MOESM7_ESM.jpg]

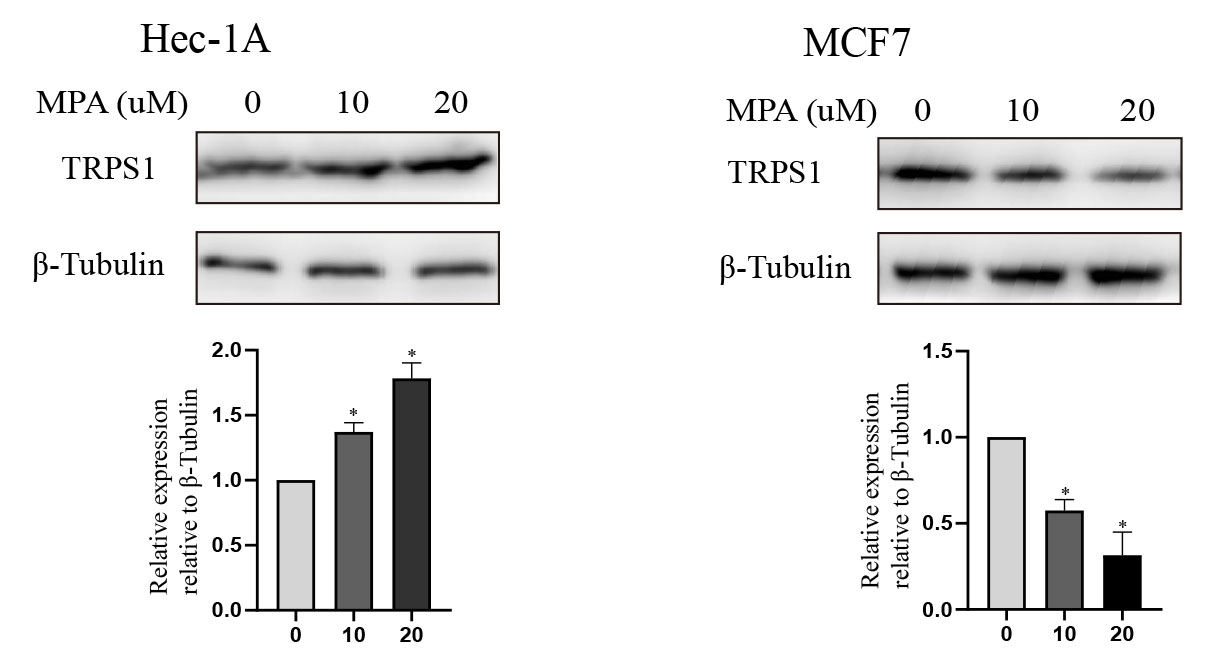

Supplement: Supplementary file 8 — Supplementary Figure8 [file 41420_2023_1484_MOESM8_ESM.jpg]

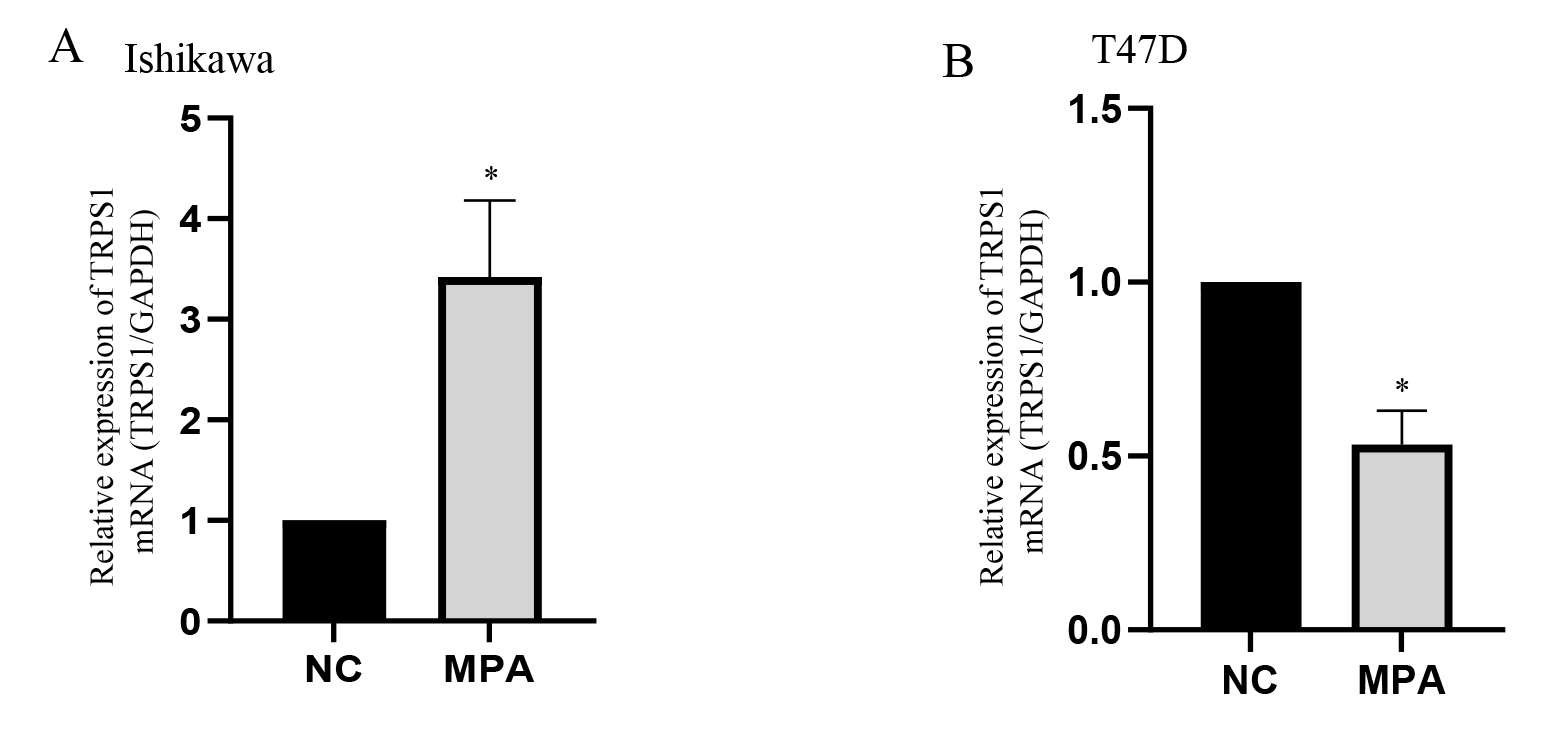

Supplement: Supplementary file 9 — Supplementary Figure9 [file 41420_2023_1484_MOESM9_ESM.tif]
