# Supplementary material for: TRPS1 regulates the opposite effect of progesterone via RANKL in endometrial carcinoma and breast carcinoma
Source: Cell Death Discov. 2023 Jun 21;9:185. doi: 10.1038/s41420-023-01484-0 (PMC10284899; doi:10.1038/s41420-023-01484-0)
Supplement: Supplementary file 10 — Supplementary Figure legends [file 41420_2023_1484_MOESM10_ESM.docx]

Supplementary Figure 1. Alterations in gene enrichment after MPA treatment in EC and BC cells, respectively. A. GO and KEGG pathway functional enrichment analysis of differently expressed genes in MPA-treated EC and BC cells, respectively. B. Differential genes were analyzed by the website of Metascape and visualized by boxplots and networks, respectively.

Supplementary Figure 2. The summary of cross-cancer alterations for relevant potential genes in EC and BC. A. The cross-cancer histogram for RANKL in 373 UCEC patients of TCGA database was conducted by cBioPortal database. The green color represents mutation and the blue represents deletion. The frequency of RANKL mutation was less than 2%. The aberrant expression threshold was defined as z-score ± 2.0 from the TCGA RNA Seq V2 data. B. The status of genetic alterations of RANKL, PR, TRPS1, HDAC2 in 373 uterine corpus endometrial carcinoma (UCEC) patients of TCGA database. The red color represents amplification, blue represents deep deletion, pink represents mRNA up-regulation and light blue represents mRNA down-regulation. Genetic alterations were found in 48 of 373 UCEC patients (13%). The aberrant expression threshold was defined as z-score ± 2.0 from the TCGA RNA Seq V2 data. This oncoprint was conducted by cBioPortal database. C. The status of genetic alterations of RANKL, PR, TRPS1, HDAC2 in 1756 patients/1918 breast cancer samples of TCGA database. The relevant genes are altered in 19 of queried patients.

Supplementary Figure 3. Efficiency verification of cell transfection. A. RANKL was overexpressed by transfecting with the plasmid for 48h in Ishikawa and T47D cells, total protein and RNA were extracted for western blot and RT-PCR analysis, respectively. B. The transfection efficiency of siRANKL was evaluated by western blot and RT-PCR assays in Ishikawa and T47D, respectively.

Supplementary Figure 4. The involvement of RANKL in the effect of MPA on cancer cells. A. HEC-1A and MCF7 cells were treated with the indicated doses of MPA for 48 h and the expression of RANKL related protein was detected by immunoblotting. B-C. CCK8 assay was performed to evaluate cell proliferation and flow cytometry was conducted to detect the percentage of apoptotic cells in HEC-1A and MCF7cells, respectively.

Supplementary Figure 5. The critical role of RANKL in the opposite regulation of MPA in endometrial cancer and breast cancer cells. A. Cell growth and apoptosis was respectively measured by CCK8 assay and Annexin V‐FITC/PI staining after RANKL knockdown in Ishikawa. B. After transfection of RANKL plasmids in T47D cells with or without using MPA, the viability and the percentage of apoptotic cells was measured by CCK8 assays and flow cytometry analysis, respectively. *p < 0.05.

Supplementary Figure 6. The correlations between PR and the traditional members of GATA family in EC and BC, respectively. Among the traditional GATA molecules, PR was negatively correlated with GATA3, the p-value was 0.044, and there was no statistically significance between PR and other members in endometrial cancers. While in BC, PR was positively correlated with GATA1 and GATA3, negatively correlated with GATA6.

Supplementary Figure 7. FISH tests were performed on all cancer cases to evaluate TRPS1 and PR fusions.

Supplementary Figure 8. The regulatory effect of MPA on TRPS1 was demonstrated in other cell lines. HEC-1A and MCF7 cells were incubated with a gradient of MPA for 48 h and then harvested for western blotting, and the expression of TRPS1 was further quantified by densitometry. *p < 0.05.

Supplementary Figure 9 The transcription levels of TRPS1 mediated by MPA in EC and BC cells, respectively. A. MPA treatment induced the transcription of TRPS1 in Ishikawa cells. B. Treated with MPA led to the declined transcription of TRPS1 in T47D cells. *p < 0.05.

Supplementary material 1. This material contains the 2756 differentially expressed genes (DEGs) upon MPA treatment by RNA-seq in Ishikawa.

Supplementary material 2. This material contains the 2811 DEGs after MPA treatment by RNA-seq in T47D cells.

Supplementary material 3. The 108 common DEGs (downregulated in EC vs upregulated in BC) and 123 common genes (upregulated in EC vs downregulated in BC) were listed.

Supplementary material 4. Motif analysis (Homer) of ChIP-seq assay and the top motifs were shown.
