# Supplementary material for: TRPS1 regulates the opposite effect of progesterone via RANKL in endometrial carcinoma and breast carcinoma
Source: Cell Death Discov. 2023 Jun 21;9:185. doi: 10.1038/s41420-023-01484-0 (PMC10284899; doi:10.1038/s41420-023-01484-0)
Supplement: Supplementary file 15 — Supplementary Tables [file 41420_2023_1484_MOESM15_ESM.docx]

**Supplementary Table 1. The sequences of siRNA**

| Name | Sequence (5’ to 3’) |
| --- | --- |
| siRANKL-1 | GGAUGGCUCAUGGUUAGAUTT |
| siRANKL-2 | CGGAUCAGGAUGCAACAUATT |
| siPR-1 | CCACCACGGUGAUGGAUUUTT |
| siPR-2 | CCGCCCGCUCUAAAGAUAATT |
| siPR-3 | CCACUGAUCAACCUGUUAATT |
| sicon | UUCUCCGAACGUGUCACGU |
| siTRPS1-1 | GUCCCUUGAAUGUAGUAAATT |
| siTRPS1-2 | GCACACAGCUGCUACAAAUTT |
| shTRPS1-1 | CCGGCTGAGGTCCTGACAAGCGATACTCGAGTATCGCTTGTCAGGACCTCAGTTTTT |
| shTRPS1-2 | CCGGGAGGTCCTGACAAGCGATAACCTCGAGGTTATCGCTTGTCAGGACCTCTTTTT |

**Supplementary Table 2. Sequences of primers used for the amplification of target genes**

| Name |  | Primer nucleotide sequence (5’ to 3’) |
| --- | --- | --- |
| TRIM29 | Forward: | GCACCGGACACCATGAAGA |
|  | Reverse: | GGAGACGAGGGCTGGTATGA |
| BMF | Forward: | CATCAAGCAGAGCACCAGCA |
|  | Reverse: | CCATTCAAAGCAAGGTTGTGC |
| ANXA1 | Forward: | CTAAGCGAAACAATGCACAGC |
|  | Reverse: | CCTCCTCAAGGTGACCTGTAA |
| NFKBIA | Forward: | ATGTGGACGACCGCCACGACA |
|  | Reverse: | ATGGCCAAGTGCAGGAACGAGTC |
| TESC | Forward: | CCTACCATTCGGAACCTGCG |
|  | Reverse: | AGCTCCTCGACCACATTTCG |
| RANKL | Forward: | GCTTGAAGCTCAGCCTTTTGCTCAT |
|  | Reverse: | GGGGTTGGAGACCTCGATGCTGATT |
| PR | Forward: | ATCCTACAAACACGTCAGTGGGCA |
|  | Reverse: | ACTGGGTTTGACTTCGTAGCCCTT |
| TRPS1 | Forward: | ATGACACTCCTGTTGGGTACT |
|  | Reverse: | CGTGCTGCTTGCCATAATGTT |
| GAPDH | Forward: | ACCCAGAAGACTGTGGATGG |
|  | Reverse: | TCTAGACGGCAGGTCAGGTC |

**Supplementary Table 3. The sequences for FISH assay**

| Name | Sense (5'-3') |
| --- | --- |
| PGR | GGTCCTGCGTCTTTTCGTCGGAGGGG |
| TRPS1 | GGCATGACCGTCCTCTTCGCCGTTGG |

**Supplementary Table 4.** The primers for ChIP-PCR and Luciferase_assay

| Name |  | Sequence (5’ to 3’) |
| --- | --- | --- |
| RANKL_promoter_PR_binding | Forward: | CTGATATCCATGGAAGACTGGTTCCA |
|  | Reverse: | CAAGTGTACAAGAGGATGTGTGTA |
| RANKL_enhancer_PR_binding | Forward: | GTCATAATATTATATTGCTGGCTTGGC |
|  | Reverse: | ATTCCCCTGGCCTATTAGTCATGAAA |
| Luciferase_assay | Forward: | TTGGAGTACTAGTAGAAATGTCATATGAAGAACTCT |
|  | Reverse: | ATATCCTTGCATACAAATTCCTTGCAAATTAT |
