# Supplementary material for: TRPS1 regulates the opposite effect of progesterone via RANKL in endometrial carcinoma and breast carcinoma
Source: Cell Death Discov. 2023 Jun 21;9:185. doi: 10.1038/s41420-023-01484-0 (PMC10284899; doi:10.1038/s41420-023-01484-0)

Figure 2E

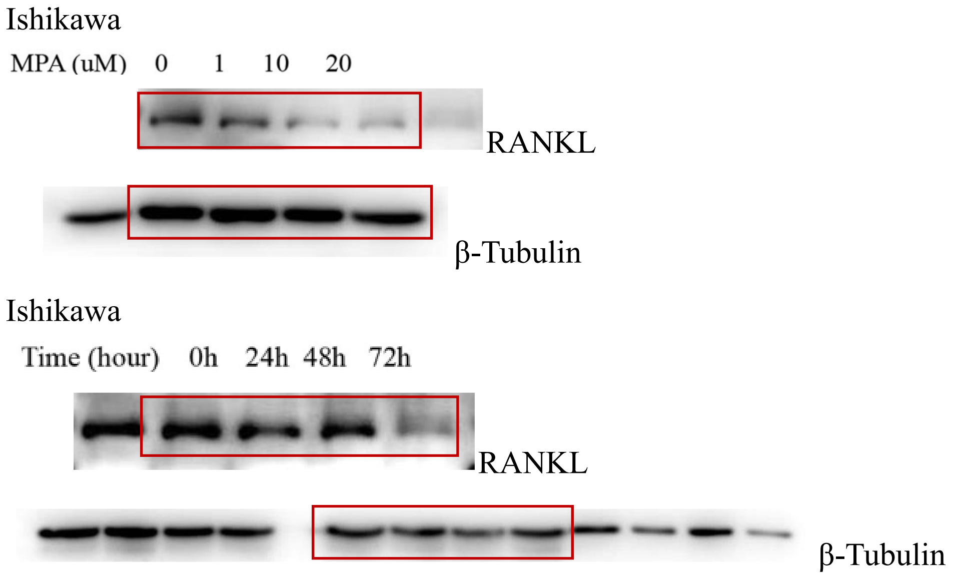

Figure 2F

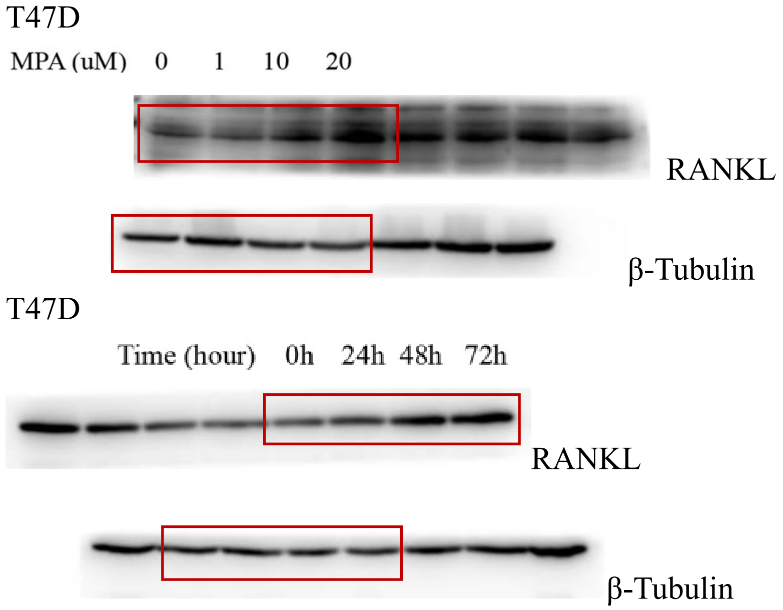

Figure 3C

Ishikawa

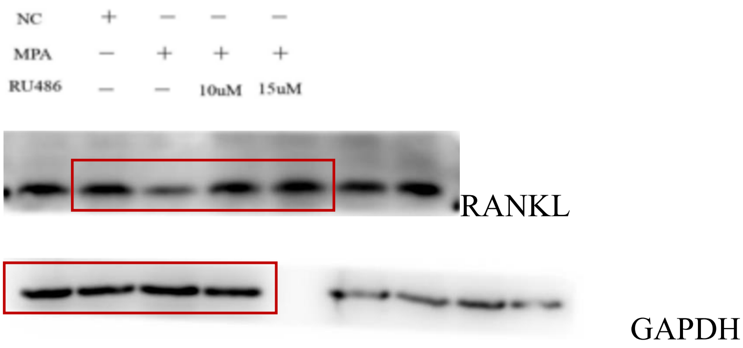

T47D

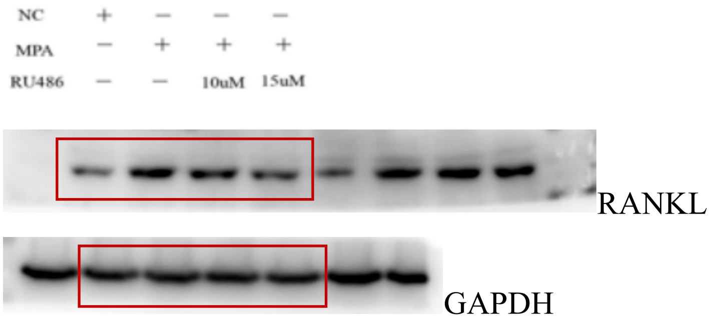

Figure 3D

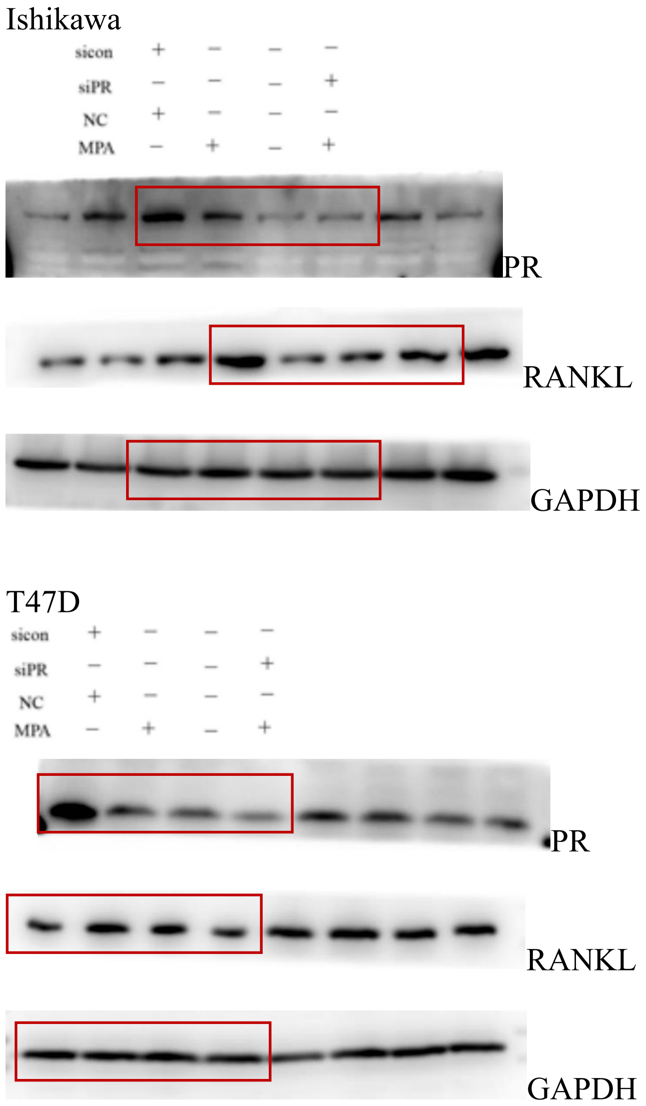
Ishikawa
sicon
siPR
NC
MPA
PR
RANKL
GAPDH
T47D
sicon
siPR
NC
MPA
PR
RANKL
GAPDH

Figure 4H

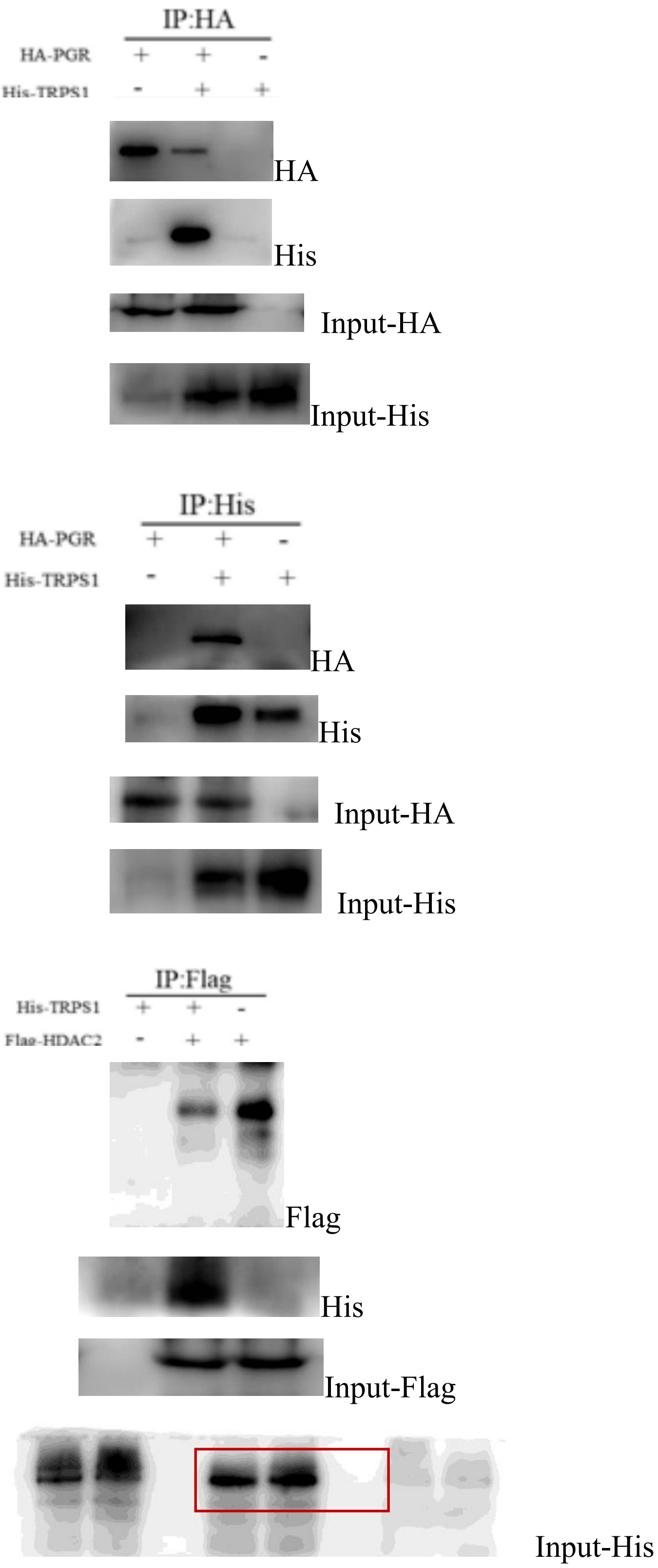
IP:HA
HA-PGR + + -
His-TRPS1 - + +
HA
His
Input-HA
Input-His
IP:His
HA-PGR + + -
His-TRPS1 - + +
HA
His
Input-HA
Input-His
IP:Flag
His-TRPS1 + + -
Flag-HDAC2 - + +
Flag
His
Input-Flag
Input-His

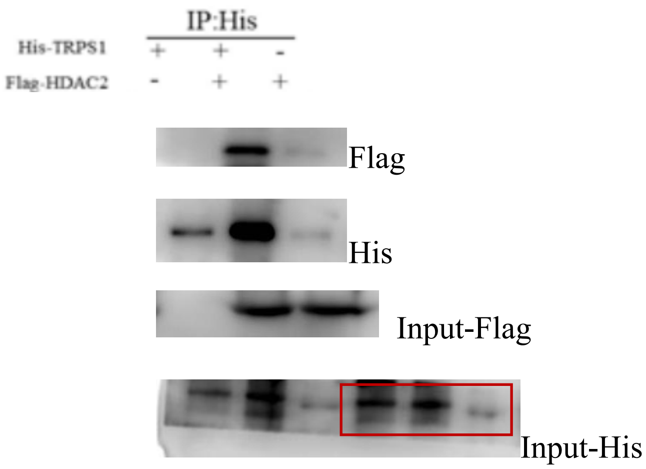
IP:His
His-TRPS1 + + -
Flag-HDAC2 - + +
Flag
His
Input-Flag
Input-His

Figure 5B

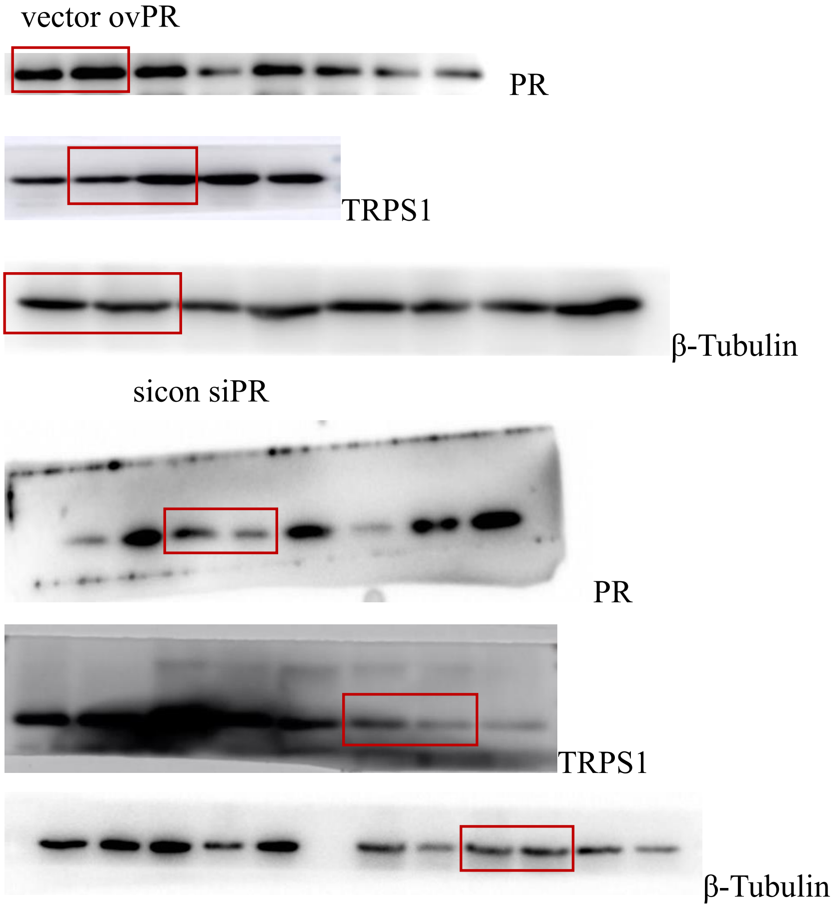

Figure 5C

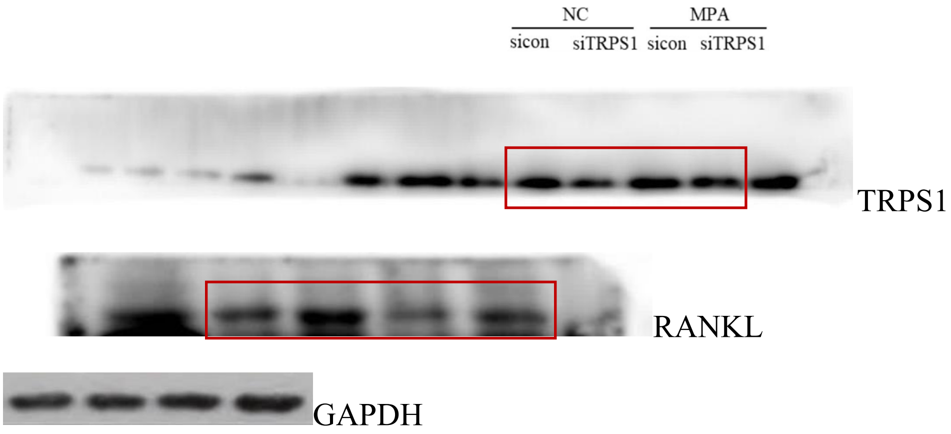

Figure 5D

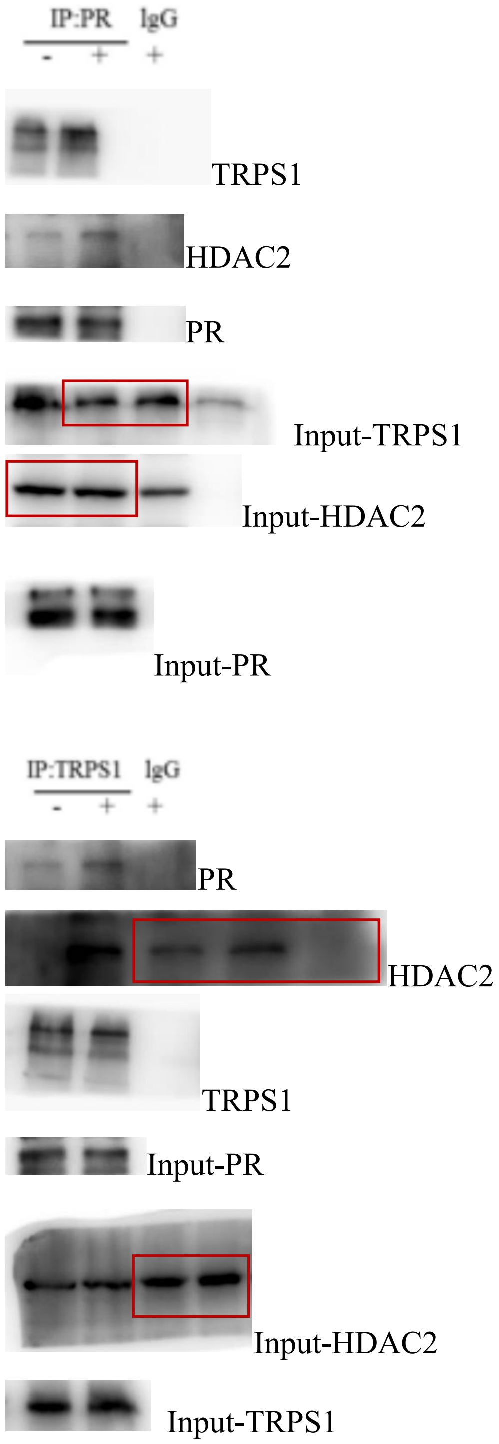
IP:PR
IgG
- + +
TRPS1
HDAC2
PR
Input-TRPS1
Input-HDAC2
Input-PR
IP:TRPS1
IgG
- + +
PR
HDAC2
TRPS1
Input-PR
Input-HDAC2
Input-TRPS1

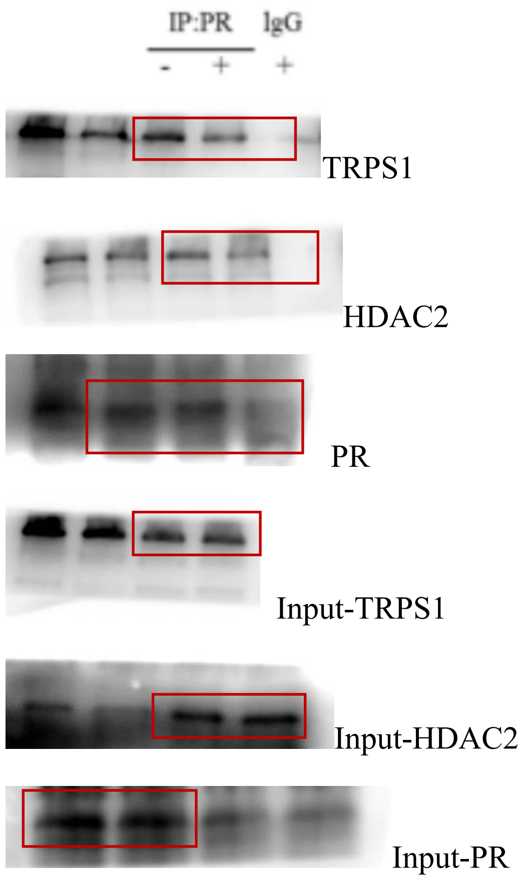
IP:PR
lgG
- + +
TRPS1
HDAC2
PR
Input-TRPS1
Input-HDAC2
Input-PR

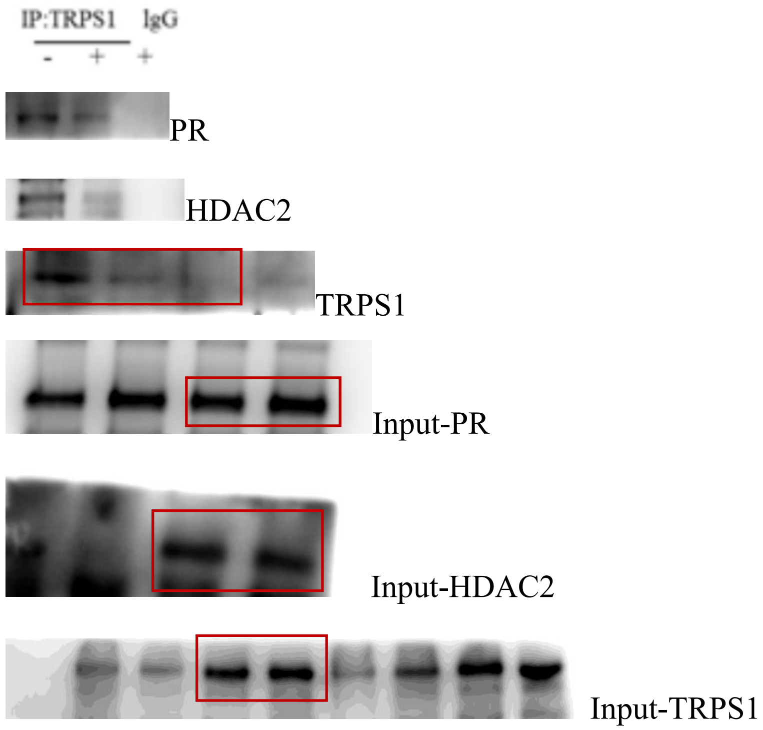
IP:TRPS1
lgG
- + +
PR
HDAC2
TRPS1
Input-PR
Input-HDAC2
Input-TRPS1

Figure 6A

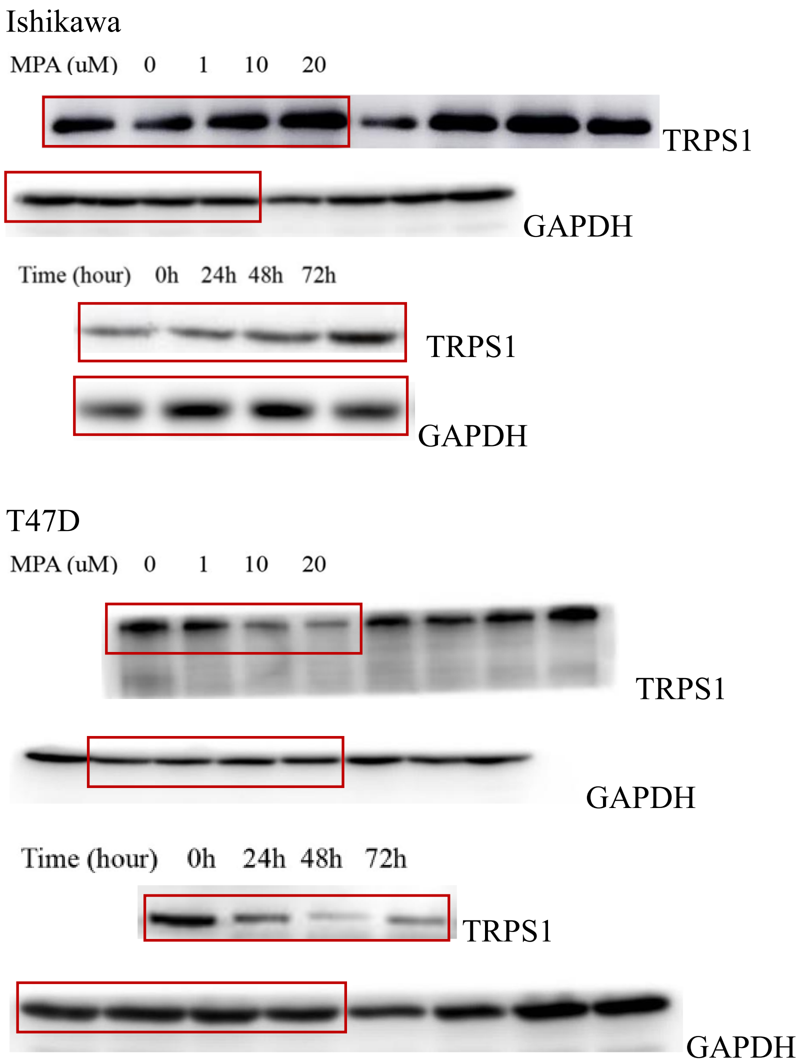
Ishikawa
MPA (uM) 0 1 10 20
TRPS1
GAPDH
Time (hour) 0h 24h 48h 72h
TRPS1
GAPDH
T47D
MPA (uM) 0 1 10 20
TRPS1
GAPDH
Time (hour) 0h 24h 48h 72h
TRPS1
GAPDH

Supplementary Figure3A

Ishikawa

vector ovRANKL

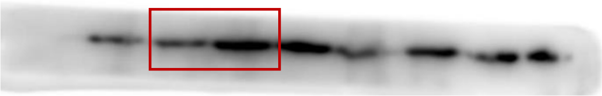

RANKL

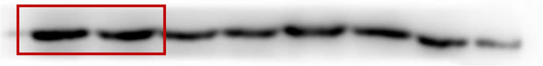

GAPDH

T47D

vector ovRANKL

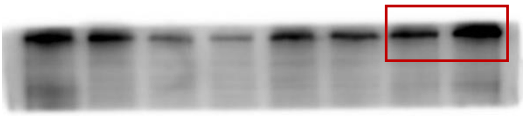

RANKL

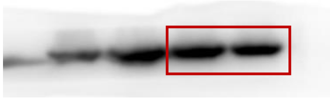

GAPDH

Supplementary Figure3B

Ishikawa

siCon siRANKL-1 siRANKL-2

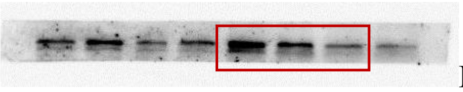

RANKL

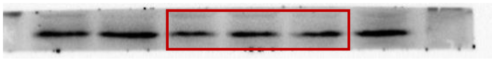

GAPDH

T47D

siCon siRANKL-1 siRANKL-2

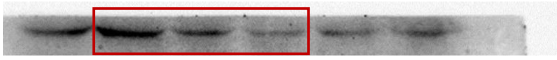

RANKL

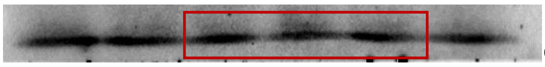

GAPDH

Supplementary Figure4

HEC-1A

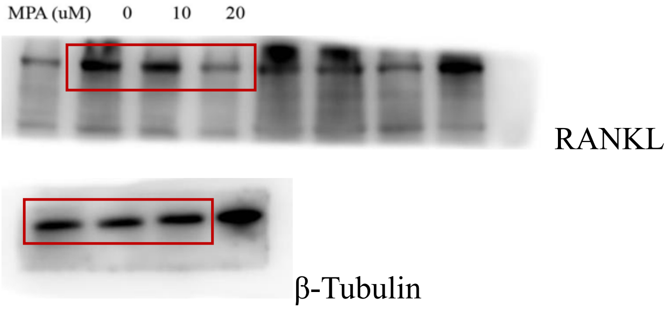

MCF-7

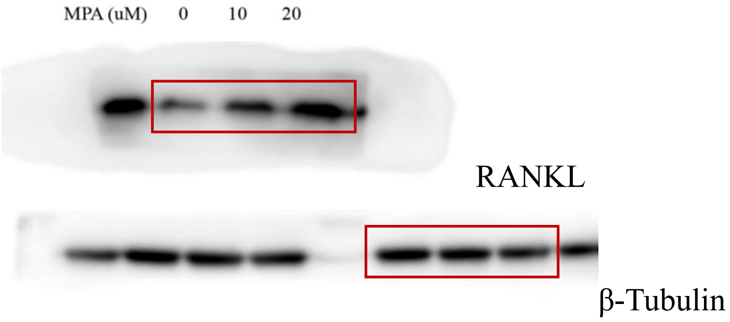

Supplementary Figure8

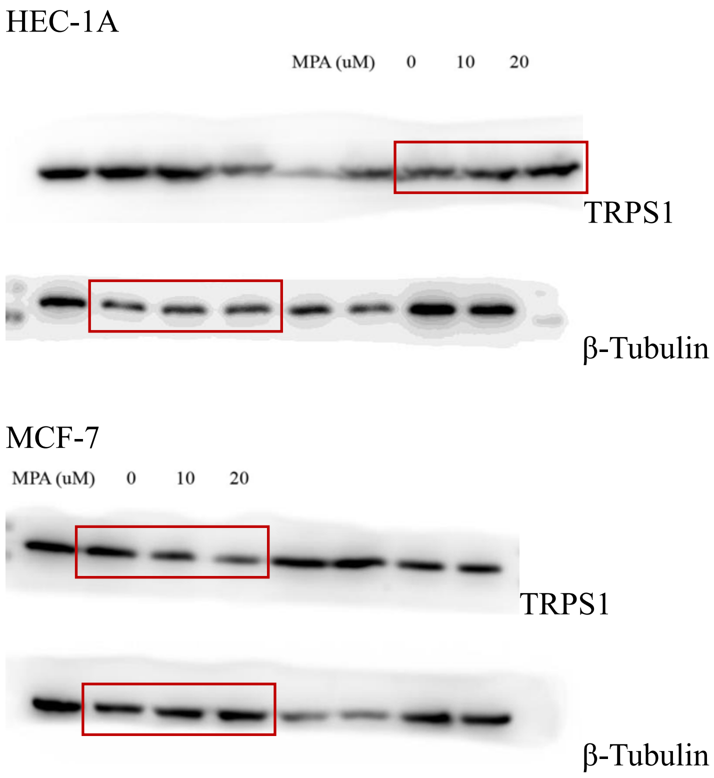

Supplement: Supplementary file 16 — Original Data File [file 41420_2023_1484_MOESM16_ESM.pdf]
